# Sustainably developing global blue carbon for climate change mitigation and economic benefits through international cooperation

**DOI:** 10.1038/s41467-023-41870-x

**Published:** 2023-10-02

**Authors:** Cuicui Feng, Guanqiong Ye, Jiangning Zeng, Jian Zeng, Qutu Jiang, Liuyue He, Yaowen Zhang, Zhenci Xu

**Affiliations:** 1 Ocean College, Zhejiang University, Zhoushan, China; 2 Donghai Laboratory, Zhoushan, China; 3 Key Laboratory of Marine Ecosystem Dynamics, Second Institute of Oceanography, Ministry of Natural Resources, Hangzhou, China; 4 Hainan Institute of Zhejiang University, Sanya, China; 5 Zhejiang Institute of Hydraulics & Estuary, Hangzhou, China; 6 Department of Geography, The University of Hong Kong, Hong Kong, China

**Keywords:** Ocean sciences, Climate change, Sustainability, Climate-change mitigation, Water resources

## Abstract

Blue carbon is the carbon storage in vegetated coastal ecosystems such as mangroves, salt marshes, and seagrass. It is gaining global attention as its role in climate change mitigation and local welfare growth. However, a global assessment on the long-term spatiotemporal sustainable development status of blue carbon has not been conducted, and the relations among blue carbon ecosystems, driving forces for climate change mitigation, and socioeconomic interventions for development capacity on a global scale are still unclear. Here, we constructed a blue carbon development index (BCDI), comprising three subsystems: driving force, resource endowment, and development capacity, to assess the sustainable development level of 136 coastal countries’ blue carbon over 24 consecutive years and explore the relationship among subsystems. We further propose a cooperation model to explore the feasibility of global blue carbon cooperation and quantify benefit allocation to specific countries. The results showed an upward trend in BCDI scores with variations in regional performance over the past two decades, and we found a positive correlation between development capacity and blue carbon resource endowment. Based on the scenario simulations of global cooperation, we found that coastal countries could improve the global average BCDI score, add 2.96 Mt of annual carbon sequestration, and generate $136.34 million in 2030 under Global Deep Cooperation scenario compared with the Business-As-Usual scenario.

## Abstract (web summary)

Sustainable development of blue carbon has increased globally over the past two decades. Global cooperation could enable countries to improve blue carbon sustainable development, increase carbon sequestration, and generate up to $136.34 million in 2030 in economic benefits.

## Introduction

Mangrove, salt marsh, and seagrass, called Blue Carbon Ecosystems, have more efficient carbon storage compared to terrestrial forests^1–3^. Blue carbon ecosystems cover 0.2% of the ocean while composing 50% of the carbon burial of the marine sediments^4^. Blue carbon ecosystems have gained increasing attention worldwide as indispensable nature-based solutions for climate change mitigation. The Intergovernmental Oceanographic Commission of the United Nations Educational, Scientific, and Cultural Organization (IOC-UNESCO), the International Union for Conservation of Nature (IUCN), and Conservation International (CI) jointly established the Blue Carbon Initiative (BCI)^5,6^. And then, the 2013 Supplement to the *2006 IPCC Guidelines for National Greenhouse Gas Inventory: Wetlands*^7^ was published, and blue carbon was officially adopted as a climate change solution gradually^8^. The global carbon stock of coastal wetlands was estimated at 10447–25066 Mt in 2017^9^. In addition to carbon burial and storage, blue carbon ecosystems can also provide various ecological services, such as pollution purification, coastal disaster mitigation, and important nursery habitat provision^10–13^.

Current studies primarily focus on blue carbon sinks and storage, and some researches have explored the socioeconomics and governance of blue carbon, such as management and strategies to minimize losses^14–17^, and the assessment of blue carbon wealth through carbon social cost^18^. However, the status of the sustainable development level of blue carbon is still unknown. Moreover, the relations among blue carbon ecosystems, driving forces for climate change mitigation, and socioeconomic interventions for development capacity on a global scale remain unclear. Such information is urgently required for promoting the global conservation and restoration of blue carbon ecosystems and the sustainable development of coastal zones.

**Fig. 1:**
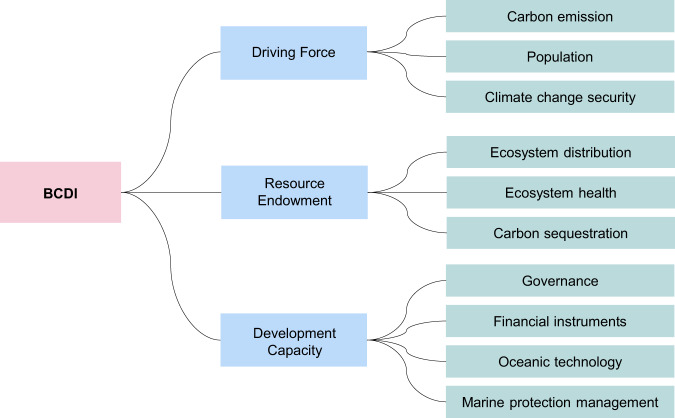
The blue carbon development index (BCDI) framework comprises three subsystems: driving force, resource endowment, and development capacity. Driving force subsystem describes drivers consisting of natural and anthropogenic elements that have positive or negative impacts^82,83^ on climate change mitigation, including carbon emissions, population, and climate change security; Resource endowment describes the ecological and environmental status of blue carbon ecosystems, covering the distribution of ecosystems (mangroves, salt marshes, and seagrass beds), ecosystem health, and carbon sequestration; Development capacity represents the capacity of socioeconomic interventions such as governance, financial instruments, oceanic technology, and marine protection management to support blue carbon development.

Here we analyze the long-term blue carbon sustainable development level of global coastal countries and adopt a global cooperation model to collectively cope with global climate change challenges and enhance the synergies between blue carbon and society. This study aims to establish a blue carbon development index (BCDI) that integrates three subsystems (driving force, resource endowment, and development capacity, Fig. 1) to assess the long-term sustainable development level of blue carbon in 136 coastal countries and explore interrelationships between subsystems. The sustainable development level of BCDI refers to the performance on conservation and restoration of blue carbon ecosystems, and the capacity and ability of socioeconomic interventions to develop blue carbon under global climate change and human impacts. Furthermore, a cooperation model was adopted to investigate the feasibility of global cooperation to increase carbon sequestration and the economic benefits of blue carbon. The results provide a sketch of the spatial and temporal sustainable development of blue carbon and a strategy to achieve ecological and economic benefits through cooperation, guiding future policy-making and international cooperation.

## Results

### Score rising over the past two decades

**Fig. 2:**
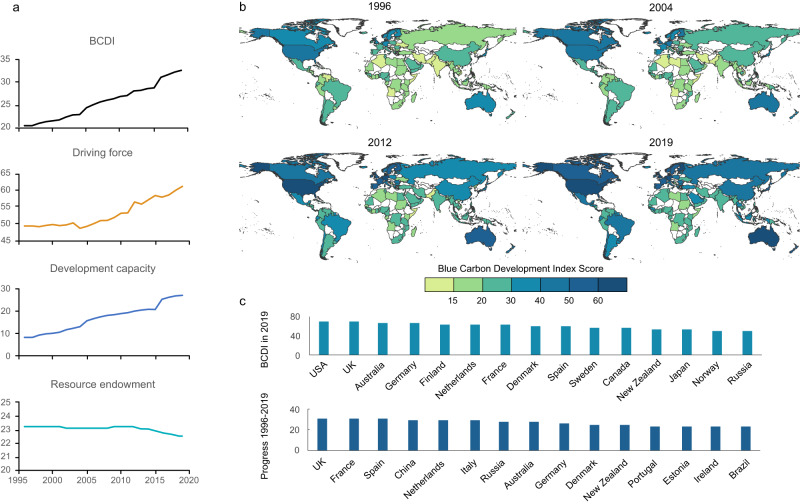
Blue carbon development index score from 1996 to 2019. **a** Global scores of BCDI and three subsystems. Higher scores indicate better performance towards BCDI and subsystems. **b** BCDI scores of countries in 1996, 2004, 2012, and 2019. Blank base map from publicly available Database of Global Administrative Areas (https://gadm.org/). **c** Countries with highest scores in 2019 and countries made the most progress from 1996 to 2019.

The BCDI score increased from 20.26 in 1996 to 32.55 in 2019, a growth of 60.69% (Fig. 2). The driving force subsystem score rose by 23.86% over a 24-year period, indicating a stronger force to stimulate the mitigation of climate change and sustainable development of blue carbon, which is primarily associated with the optimization of energy structure and the reduction in carbon emission intensity. And the development capacity score demonstrated a significant advance from 8.59 to 27.36 as a result of overall improvements in ocean governance, economic strength, technology, and marine protection. While the resource endowment exhibited a slow decline, with the score dropping from 23.23 to 22.57, this was mostly due to a reduction in the distribution area and a decline in ecosystem health. The global mangrove area decreased by 87,695.07ha^19^ and the ecosystem health score decreased^20^ between 2011 and 2013. Of the included countries, only 23.53% maintained or amplified their resource endowment scores from 1996 to 2019. Interestingly, we discovered that the subsystem scores displayed differences, while the overall BCDI rose steadily. We also drew box plots (Supplementary information Fig. S1) for the indicator data to analyze the original data distribution and found that the data of most indicators in the development capacity and resource endowment subsystems were significantly polarized, leading to low values in most countries.

Over the two decades, significant progress has been made in BCDI scores of many countries (Fig. 2). Most countries in the Americas, Asia, and Europe showed a noticeable improvement in BCDI scores, while African countries showed a slight enhancement. The United States occupies the top position among all the countries in 2019, mainly due to a fairly high score in the development capacity subsystem. In addition, countries such as United Kingdom, Australia, Germany, Finland, Netherland, France, Spain, Denmark, Sweden, Canada, and Japan also have relatively high BCDI scores. The large advance in BCDI scores for countries between 1996 and 2019 (Fig. 2(c)) was predominantly due to better performances in their development capacity and driving force subsystems. However, the increase of BCDI scores in China is mainly attributed to the rapid growth in the development capacity subsystem (Supplementary information Fig. S2). Furthermore, there are some countries with persistently low BCDI scores in our analysis, such as Angola, Haiti, Libya, Bosnia and Herzegovina, Somalia, Algeria, Bahrain and several others. Most of these countries belong to the regions of Africa and Asia. They exhibit lower scores across all three subsystems, with insufficient ocean protection and management, limited blue carbon ecosystems, and serious challenges with climate change.

### Variations between regions

**Fig. 3:**
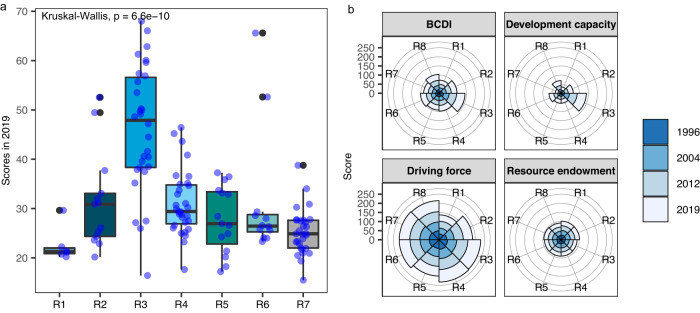
Variations between regions. **a** Boxplot of BCDI scores and Kruskal--Wallis test of different regions in 2019. There were significant differences among regions (Kruskal–Wallis test, *P*-value < 0.001). The boxplot displays the median value at the centre, while the lower and upper bounds represent the first and third quartiles. The upper and lower whiskers extend from the bounds to the largest or lowest value, but no further than 1.5 times the IQR (the distance between the first and third quartiles). Sample sizes for each region: R1 (*n* = 6), R2 (*n* = 13), R3 (*n* = 30), R4 (*n* = 31), R5 (*n* = 15), R6 (*n* = 12), and R7 (*n* = 29). **b** BCDI and subsystems scores of different regions from 1996 to 2019. R1-Central and Southern Asia, R2-Eastern and South–Eastern Asia, R3-Europe and Northern America, R4-Latin America and the Caribbean, R5-Northern Africa and Western Asia, R6-Oceania, R7-Sub-Saharan Africa, R8-Global average. The division of regions is based on the UN’s regional groupings (https://unstats.un.org/sdgs/indicators/regional-groups).

Regions around the world exhibit variations in the scores of BCDI and subsystems (Fig. 3). Europe and North America (ENA) and Oceania scored higher in BCDI than other regions, while the other five generally scored lower than the worldwide average. There were significant differences in BCDI grades among regions (Kruskal–Wallis test, *P* < 0.001; Fig. 3). Eastern and South–Eastern Asia (ESA) scored far below the score of Oceania prior to 2012, after which they had noticeably increased and moved closer to it. It is assumed that the trigger for this phenomenon was the improvement in the development capacity subsystem. Central and Southern Asia (CSA) and Eastern and South–Eastern Asia (ESA) possessed excellent resource foundations of blue carbon. Although CSA appeared rich in resources endowment of blue carbon, the driving force and development capacity subsystems were inadequately developed. The ESA also performed relatively poorly in the driving force subsystem. In addition, with even performances in all three subsystems, the Oceania region was the most balanced of all regions. ENA displayed the highest scores in the development capacity, stemming from complete management mechanisms and adequate capital guarantees. For Latin America and the Caribbean (LAC) and Sub-Saharan countries, development capacity showed to be the limit to BCDI performance, and it is necessary to improve social interventions to support blue carbon.

### Relations between development capacity and resource endowment

Our study reveals a statistically significant positive correlation between development capacity and resource endowment in 136 countries (*P* < 0.001. Supplementary information Fig. S3), indicating that countries with better marine management and protection achieved higher resource endowments in blue carbon ecosystems. Countries with higher development capacity scores indicate that these countries have better environmental management, higher marine funding, and more cutting-edge science and technology for the protection and restoration of blue carbon ecosystems. Recent research has reported that the increasing conservation efforts have led to a reduction in anthropogenic loss of mangroves^21^, and that economic growth has shifted from negatively affecting mangroves to promoting mangrove expansion over the past decade^22^. Currently, global policy support, management, and conservation practices on the oceans have been increasing, which can have positive influences on blue carbon ecosystems^23,24^.

### Global cooperation enhances carbon sequestration and economic benefits

**Fig. 4:**
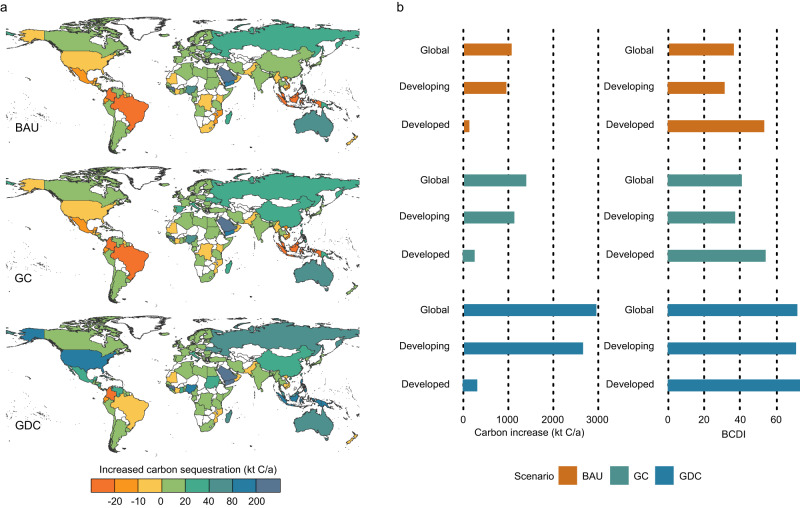
Analysis of carbon sequestration increase and BCDI under cooperation scenarios. **a** Carbon sequestration increase allocation in 2030 under three scenarios compared to 2019 at national scale. Blank base map from publicly available Database of Global Administrative Areas (https://gadm.org/). **b** Carbon sequestration increase allocation and BCDI score of global, developed, and developing countries.

We set up three scenarios to reveal the effects of global cooperation on carbon sequestration and BCDI scores in 2030: Business-As-Usual (BAU), Global Cooperation (GC), and Global Deep Cooperation (GDC). In the cooperation scenarios, countries with higher development capacity scores could assist those with lower scores to manage and restore blue carbon ecosystems, leading to an increase in annual carbon sequestration. The results showed that under the GC scenario, the world could potentially increase carbon sequestration by 1.39Mt from 2019 levels (Fig. 4), which is a 29.56% improvement compared to the BAU scenario (1.07Mt). The GDC scenario demonstrated even greater potential for annual carbon sequestration growth at 2.96Mt, an improvement of 177.12% compared to BAU scenario. Moreover, we found that $136.34 million in economic benefits could be generated globally under the GDC scenario.

Cooperation can enable developing and developed countries to achieve win-win results. Developed countries can expand the scale of emissions reduction and further take on the responsibilities of climate change mitigation, while developing countries have chances to promote the resource endowment of blue carbon and improve ocean management and marine technology. According to our results, BCDI scores of developing countries could increase by 17.50% and 125.28% in the GC and GDC scenarios, respectively.

The increases in carbon sequestration under cooperation scenarios mainly originate in developing countries. Saudi Arabia, Guinea, Yemen, Australia, Nigeria, Papua New Guinea, and the Philippines could increase the most carbon sequestration under BAU and GC scenarios. Notably, the United States, Indonesia, and Malaysia could substantially enhance their carbon sequestration in the GDC scenario (Fig. 4). In the BAU and GC scenarios, the blue carbon sequestration in Indonesia and Malaysia suffers severe losses but is greatly enhanced in the GDC scenario. The United States, on the other hand, has a high development capacity score and is able to provide support to other countries, increasing 146.37 kt of blue carbon sequestration in GDC scenario compared to GC scenario. Although developed countries show less increase in the quantity of carbon sequestration than developing countries, their benefits from cooperation are considerable. Under the GDC scenario, developed countries could obtain more blue carbon through their advantages in development capacity, which increases by 23.60% relative to the GC scenario.

## Discussion

This study illustrated the spatial and temporal development status of blue carbon on global and national scales and explored the relations between socioeconomic factors and blue carbon by establishing the blue carbon development index (BCDI). Our analysis showed an overall increase in BCDI, with variations in performance by different regions, and that the related socioeconomic interventions for development capacity have a significantly positive correlation with the resource endowment of blue carbon ecosystems. Thus, the proposed global cooperation model in this study could provide a feasible way to enhance the local ocean management and promote the conservation and restoration of blue carbon ecosystems.

We observed an upward trend in BCDI, driven by the combined effects of three subsystems, with development capacity exerting the most significant influence. The increasing score of development capacity indicates a positive shift towards better policy making and conservation management that contributes to the sustainable development of blue carbon. The increase in score of development capacity outpaces the decrease in score of resource endowment of the blue carbon ecosystems, resulting in an overall upward trend for BCDI. The resource endowment displayed a decreasing trend, primarily due to human activities and extreme weather events, leading to a shrinkage in the spatial distribution of coastal blue carbon ecosystems^21,25,26^ and a deterioration in ecosystem health^27,28^. Factors such as, aquaculture, agriculture, human settlement, and reclamation have contributed to the reduction of blue carbon ecosystems in some developing countries, especially the agricultural countries in Southeastern Asia^29,30^. However, ocean management and protection have the potential to positively enhance the resource endowment of blue carbon ecosystems and have already struggled to combat the deterioration of blue carbon ecosystems. In fact, owing to the increased awareness, improved conservation, and enhanced restoration efforts, the current downward trend of blue carbon ecosystems has already slowed down^21,22^. If conservation efforts for blue carbon ecosystems are adopted and strengthened consistently, it is possible to stabilize and even reverse decreasing trends in resource endowment, leading to future potential recovery^23,31^. For instance, China successfully reversed the trend of mangrove forest loss through mangrove restoration and protection after suffering significant losses as a result of marine industry development in the last century^32^.

We also found variations in the blue carbon sustainable development level across regions, which could be mitigated by feasible international cooperation. For example, Asia is a region with strong resource endowments^33,34^ but with greater carbon reduction pressures and less development capacity. Weak economies, inadequate coverage of marine protected areas, and deficiencies in marine policy and management are major disadvantages for countries in Asia in improving the sustainable development level of blue carbon. Blue carbon ecosystems are currently facing serious damage and loss in some agricultural countries in Asia^29,35^. Latin America and the Caribbean (LAC) and Sub-Saharan Africa also have both relatively low levels of development capacity. Countries in Asia, LAC, and Sub-Saharan Africa can actively participate in blue carbon projects to enhance their development capacity through cooperation with developed countries. They can also add their nationally determined contributions (NDCs) and promote economic progress through blue carbon trading^8,36^. Countries in Europe and Northern America (ENA) and Oceania could help other countries through cooperation. At the same time, these countries providing assistance can expand their international influence and gain additional blue carbon benefits, thus achieving a win-win situation for both parties. A total of $136.34 million of blue carbon economic benefits are generated globally in the GDC scenario, while the corresponding cost is $82.79 million (Supplementary information Fig. S4). As an emerging field, there are some ongoing blue carbon cooperation programs, such as the Blue Carbon Initiative established by Conservation International (CI) and the International Union for Conservation of Nature (IUCN), the International Blue Carbon Partnership^37^ launched by Australia, and China has also released its blue carbon cooperation program with Southeast Asian countries. These experiences can lay a foundation for extending blue carbon cooperation to a global scale to promote better blue carbon sustainable development.

Promoting the overall sustainable development level of blue carbon could also bring co-benefits that contribute to the social well-being and livelihoods of local coastal communities. Blue carbon ecosystems play a critical role in shielding coastal regions from floods^38^ and tropical storms^39^, as well as supporting long-term gains of fisheries^40^. Besides, blue carbon ecosystems could also provide recreational opportunities such as fishing, swimming, sun-bathing, boating, bird-watching, and diving, enhancing the overall local well-being^41,42^. Protecting and restoring blue carbon ecosystems could enhance local resilience in coping with climate change and align with multiple Sustainable Development Goals (SDGs) of the United Nations^31^. The local government can take proactive steps such as implementing protection and restoration measures, encouraging international blue carbon trading, and building mechanisms for communication on management and technology with international organizations and other nations. International organizations should improve the global cooperation platform and provide targeted financial and technical support to local governments in need. It is also essential to raise awareness among community residents through cooperation and encourage them to reduce damage to blue carbon by controlling agriculture, fishing, and other activities^43^.

It’s also important to extend and adapt BCDI system to local scales in the future once local-scale data becomes available, paving the way for local government and the community to implement more efficient socioeconomic interventions for improving the sustainable development level of blue carbon both at the local and national level. By incorporating the local-scale data, researchers may be able to identify the reasons causing strong or weak performance on BCDI more precisely, so as to facilitate local management practices^44,45^ and promote the blue carbon cooperation more effectively. To implement BCDI framework at local levels in future research, adjustments to the current BCDI indicator system can be necessary. Firstly, we should downscale the indicators and incorporate more specific indicators that could be monitored at the local level, e.g., local blue carbon projects, local financial capacity, and local blue carbon vegetation species. Secondly, it is crucial to take into account the social impact and response of indigenous residents and communities on blue carbon management, such as the impacts on their livelihoods, their awareness of blue carbon, and their participation in policymaking.

Currently, management and social aspects are becoming important themes in blue carbon science^12^, and blue carbon initiatives at the national and local scales have been proposed^46,47^. To facilitate the management of blue carbon, clear and measurable objectives must be defined at national and local scales. An effective management framework should take into account regional livelihoods and integrates the dimensions of governance, finance, and technology^48^. In addition, a clear benefit-sharing mechanism is needed to ensure equitable distribution of blue carbon benefits, and ecosystem services should be integrated as well^46^.

We acknowledge the presence of uncertainty in our indicators and model settings due to the use of various types of global datasets from different sources and literature. We employed proximity interpolation methods to fill in incomplete data from some regions. This can be improved by dataset accuracy increases. Moreover, while we focus on the carbon sink function of blue carbon ecosystems, we need to pay attention to their additional ecosystem services^46^. However, ecosystem services were not considered in the indicators of this study due to the lack of data at the national level across the globe, and future work could include additional ecosystem services, such as storm protection, habitat provision, and pollution uptake, in the indicator system once the national data becomes available. We must also note that blue carbon is not the only nature-based solution; there are also other solutions such as forests, peatlands, and grassland^49,50^, and achieving the Paris Agreement goals requires natural-based solutions and a combination of other approaches: energy structure optimization^51^, the establishment of carbon trading markets^52^, and the expansion of carbon capture and storage^53^.

## Methods

### Index system

Based on existing global datasets on the distribution of mangroves, salt marshes, and seagrasses, we identified 136 countries with blue carbon ecosystem distributions. We then constructed an indicator system and evaluated the sustainable development level of blue carbon in global countries. We established the BCDI indicator system framework based on three subsystems (Fig. 1): driving force, resource endowment, and development capacity. The indicator system includes 18 indicators (Supplementary information Table S1).

### Comprehensive assessment

The comprehensive scores of each country and region were acquired using a weighted calculation of each indicator. We employed the principal component analysis (PCA) method^54^ to determine the weights of indicators (Supplementary information Table S1). The principal component analysis (PCA) is a classic multivariate statistical method for dimensionality reduction of unsupervised, high-dimensional data while maintaining as much of the original information as possible^54,55^. PCA can be implemented by diagonalizing the weighted covariance matrix, where we can obtain the associated weights for each indicator. The weights obtained through principal component analysis (PCA) are based on the objective variation characteristics of the data^56^. The weights obtained through PCA specify the uniqueness of subsystems and reduce collinearity, which determines that one subsystem cannot be easily substituted by another.

Subsystem scores were obtained as follows:

1 $${I}_{k}=\frac{1}{{w}_{k}}\,\mathop{\sum }\limits_{j=1}^{n}{w}_{j}*{y}_{i,\,j}$$

where ${I}_{k}$ is the score of subsystems, $k$ represent driving force, resource endowment, and development capacity,$\,{w}_{j}$ is the weight of indicators, ${y}_{i,\,j}$ is standardized value. The weight ${w}_{j}$ for each indicator was obtained by normalizing the PCA weight matrix across all indicators from three subsystems simultaneously^57–59^. ${w}_{k}$ is the weight of a subsystem, which is the sum of ${w}_{j}$ for all indicators in the subsystem.

The calculation of BCDI score is as follows:

2 $${I}_{{BC}}=\mathop{\sum }\limits_{j=1}^{n}{w}_{k}*{I}_{k}$$

where ${I}_{{BC}}$ represents the BCDI score,$\,{w}_{k}$ is the weight of different subsystems. ${I}_{k}$ and ${I}_{{BC}}$ range from 0 to 1, with a higher value indicating better performance. The average grade of all nations is regarded as the global score for each year.

To obtain comparable indicators, the index data were standardized to ensure that the index value is between 0 and 100. The standardization of the positive and negative indicators was moderately different. This data standardization across all years and regions permits a comprehensive evaluation of the changes in indicators over time and space^60^. For positive indicators:

3 $${y}_{i,\,j}=\frac{{x}_{i.j}-{x}_{j,{lower}}}{{x}_{j,{upper}}-{x}_{j,{lower}}}\times 100$$

For negative indicators:

4 $${y}_{i,\,j}=\frac{{x}_{j,{upper}}-{x}_{i.j}}{{x}_{j,{upper}}-{x}_{j,{lower}}}\times 100$$

where ${x}_{i,\,j}$ represents the $j$ th indicator value during the $i$ th year, ${y}_{i,\,j}$ is the standardized value, ${x}_{j,{upper}}$ is the upper bound of the $j$ th indicator, and ${x}_{j,{lower}}$ is the lower bound of the ${j}$ th indicator. As for the upper bound and lower bound, we set the data points at the top 2.5th percentile and the bottom 2.5th percentile of all countries and years. By selecting the upper and lower bounds of a range of values, this strategy can avoid the influence of outliers that may distort the results and analysis. The bound selection measure has been widely employed for ranking indicator performances in articles and reports, such as research articles^61,62^, the SDGs Report^63^, and the handbook of Organization for Economic Co-operation and Development^56^.

### Linear mixed models

We utilized the linear mixed model to investigate the relationship between the blue carbon ecosystem area and the three subsystems to roughly predict future area changes. We compared the AIC of several models and selected the model whose independent variable was the development capability score (See supplementary information for details).

5 $${Rate}={\beta }_{0}+{\beta }_{1}{ln}{x}_{1}+\left(1 | {nation}\right)$$

*Rate* refers to the growth rate of the blue carbon area over the next 4 years. ${x}_{1}$ is the development capacity score, ${\beta }_{0}$ is the intercept parameter, and ${\beta }_{1}$ is the fixed effect parameter. Considering each country may vary greatly, countries are selected as random effect variables. Then we could estimate the carbon sequestrations:

6 $${{Carbon}}_{j}={Are}{a}^{(1+{{Rate}}_{j-4})}*{CRate}$$

where ${{Carbon}}_{j}$ refers to the carbon sequestration in year $j$, and ${Area}$ refers to the blue carbon ecosystem area of base year, ${Rate}$ indicates the growth rate of the blue carbon area over the previous 4 years (from linear mixed models), ${CRate}$ represents the average rate of carbon sequestration of different countries (from indicator Y5).

To explore the effects of the model, we performed some verifications. Comparing the observed and simulated values, we found both sets of values were distributed near the line, with a slope of 1. Moreover, the residual picture showed that the residual distribution was near zero with no pattern, indicating no correlation with the residual and the value of the independent variable. A Q-Q plot also verified the normal distribution of the residuals, with most residuals clustered on straight lines (see supplementary information for details).

### Cooperation modeling and scenario setting

To study future changes in the global BCDI and the feasibility of the global cooperation, we set up three scenarios for analysis: the business as usual (BAU) scenario, global cooperation (GC) scenario, and global deep cooperation (GDC) scenario. The BAU scenario applies Holt’s linear trend model to fit and predict the scores for each subsystem in >100 countries.

The GC and GDC scenarios were based on the BAU scenario, assuming that global blue carbon countries unite in cooperation. Countries with higher performance in the Development Capacity subsystem of this cooperation model could help countries with lower scores. We established a basic cooperation model, allocating the collaborative benefits of carbon sequestration at a country-specific scale according to three methods: the Equal surplus division value method, the Equal allocation of non-separable costs method, and the separable cost remaining benefit method, and chose the most proper method. In cooperation, the benefits to each member are greater than they would receive if they developed individually. To quantitatively set up cooperation scenarios, we employed and optimized a methodology proposed by Zhao et al.^64^, which assigned different technology levels to members of cooperation. Continued quantile points of indicators were selected as the states that can be achieved with different cooperation intensities. Subsequently, building on the idea of maximizing global welfare in a cooperative game of the Integrated Assessment Model^65^, we conducted a cost-benefit analysis to identify scenarios with locally optimal solutions. Following the cost-benefit analysis and scenario selection, we proceeded with a detailed analysis of the chosen scenarios.

Holt’s linear trend model: The exponential smoothing method can predict future data changes by weighting past data. Holt’s linear trend extends the simple exponential smoothing model to predict the data containing the trend^66^, consisting of a prediction equation and two smoothing equations:

7 $${l}_{t}=\alpha {x}_{t}+(1-\alpha )({l}_{t-1}+{b}_{t-1})$$

8 $${b}_{t}=\beta ({l}_{t}-{l}_{t-1})+(1-\beta ){b}_{t-1}$$

9 $${\hat{x}}_{t+h}={l}_{t}+h{b}_{t},h=1,2,3\ldots$$

Where $t$ is the current period, $h$ is prediction step length. And ${x}_{t}$ refers to observation value in period $t$, ${l}_{t}$ is the prediction value in period $t$, ${b}_{t}$ is prediction trend. $\alpha$ refers to horizontal smoothing parameter, and $\beta$ is trend smoothing parameter.

The Separable Cost Remaining Benefit (SCRB) method^67,68^:

10 $${b}_{i}={m}_{i}+\frac{{r}_{i}}{\mathop{\sum}\nolimits_{i\epsilon U}{r}_{i}}\left[v\left(U\right)-\mathop{\sum}\limits_{i\in U}{m}_{i}\right]$$

11 $${m}_{i}=v\left(U\right)-v\left(U/i\right)$$

12 $${r}_{i}={m}_{i}-v\left(\{i\}\right)$$

The Equal surplus division value (ESD) method^69,70^:

13 $${b}_{i}=v\left(\{i\}\right)+\frac{1}{n}\left[v\left(U\right)-\mathop{\sum}\limits_{i\in U}v\left(\{j\}\right)\right]$$

The Equal allocation of non-separable costs (EANC) method^70,71^:

14 $${b}_{i}={m}_{i}+\frac{1}{n}\left[v\left(U\right)-\mathop{\sum}\limits_{i\in U}{m}_{i}\right]$$

where ${m}_{i}$ is the separable benefit, and ${r}_{i}$ is the remaining benefit of member $i$. ${b}_{i}$ indicates the final benefit allocation, U means a coalition with $n$ member, $v\left(U/i\right)$ is the benefit of coalition except $i$, and $v(U)$ represents the value of the grand coalition, $v\left(\{i\}\right)$ is the value of members in non-cooperation situations.

### Economic benefits and cost calculation through cooperation

We applied the country-level social cost of carbon (CSCC) framework from Bertram et al.^18^ and Ricke et al.^72^ to assess the economic benefits and costs under cooperation scenarios. The benefits refer to the value of redistributed carbon sequestration in cooperation scenarios compared to the business-as-usual (BAU) scenario. The costs refer to the expense of the increased carbon sequestration within country boundaries under the cooperation scenario compared to the BAU scenario.

15 $${benefit}={CSCC}*({{Carbon}}_{{coop}\,{allocation}}-{{Carbon}}_{{BAU}})$$

16 $${cost}={CSCC}*({{Carbon}}_{{coop}}-{{Carbon}}_{{BAU}})$$

where ${CSCC}$ is the country-level social cost of carbon, and ${{Carbon}}_{{coop}}$ represents the carbon sequestration of cooperation scenarios within the boundary of a country, ${{Carbon}}_{{coop\; allocation}}$ is the redistributed carbon sequestration of a country, and ${{Carbon}}_{{BAU}}$ is the carbon sequestration under the BAU scenario.

### Data sources

Data on carbon emissions were obtained from the Global Carbon Atlas^73^, and the Statistical Review of World Energy provided information on the percentage of clean energy^74^. We acquired the Climate Change Risk data from Eckstein et al.^75^ and the population density, GDP, and GDP per capita data from the World Bank. The Sci Index of Environment Science was collected from SCImago^76^, and the Corruption Perceptions Index was obtained from Transparency International^77^. We estimated the marine protected area (MPA) and management effectiveness of MPA data from Protected Planet^78^. Datasets from the literature were used to determine the areas of mangroves^19^, salt marshes^34^, and seagrass^79^. The ecosystem health data were gathered from Halpern et al.^20^, and the average carbon sequestration rates in countries were calculated based on the blue carbon ecosystem areas^19,34,79^ and carbon burial rate^1,80^. The status of accession to commitment agreements was computed from the acquisition of the Kyoto Protocol and Paris Agreement^81^. The construction status of the carbon trading market was obtained from the International Carbon Action Partnership (https://icapcarbonaction.com/en/ets). We used data from nearby years to interpolate for any missing values.

### Reporting summary

Further information on research design is available in the Nature Portfolio Reporting Summary linked to this article.

### Supplementary information

Supplementary Information

Reporting Summary

## Data Availability

All the sources of data underlying the research are available in the Supplementary Information. All other data are available from the corresponding authors upon request.

## Code availability

All computer code used in conducting the analyses summarized in this paper is available from the corresponding author upon request.

## References

[1] Mcleod E (2011). A blueprint for blue carbon: toward an improved understanding of the role of vegetated coastal habitats in sequestering CO2. Front. Ecol. Environ..

[2] United Nations Environment Programme. *Blue Carbon: The Role of Healthy Oceans in Binding Carbon.* https://wedocs.unep.org/handle/20.500.11822/7772;jsessionid=7D1A29F9BBCFF42D6F8D9EBD03F4D80A (2009).

[3] Duarte CM, Middelburg JJ, Caraco N (2005). Major role of marine vegetation on the oceanic carbon cycle. Biogeoscience.

[4] Duarte CM, Losada IJ, Hendriks IE, Mazarrasa I, Marbà N (2013). The role of coastal plant communities for climate change mitigation and adaptation. Nat. Clim. Change.

[5] Jiao N, Wang H, Xu G, Aricò S (2018). Blue carbon on the rise: challenges and opportunities. Natl. Sci. Rev..

[6] Thomas S (2014). Blue carbon: knowledge gaps, critical issues, and novel approaches. Ecol. Econ..

[7] IPCC. *2013 Supplement to the 2006 IPCC Guidelines for National Greenhouse Gas Inventories* (IPCC, 2014).

[8] United Nations Environment Programme. Blue Carbon—Nationally Determined Contributions Inventory. *Appendix to: Coastal Blue Carbon Ecosystems: Opportunities for Nationally Determined Contributions*. https://www.unep.org/ndc/resources/report/blue-carbon-nationally-determined-contributions-inventory-appendix-coastal-blue (2020).

[9] Howard J (2017). Clarifying the role of coastal and marine systems in climate mitigation. Front. Ecol. Environ..

[10] Himes-Cornell, A., Pendleton, L. & Atiyah, P. Valuing ecosystem services from blue forests: a systematic review of the valuation of salt marshes, sea grass beds and mangrove forests. *Ecosyst. Serv*. **30**, 36–48 (2018).

[11] Barbier, E. B. The value of coastal wetland ecosystem services. in *Coastal Wetlands* 2nd edn (eds. Perillo, G. M. E., Wolanski, E., Cahoon, D. R. & Hopkinson, C. S.) Ch. 27 (Elsevier, 2019).

[12] Macreadie PI (2019). The future of blue carbon science. Nat. Commun..

[13] Hossain MS, Hashim M (2019). Potential of Earth Observation (EO) technologies for seagrass ecosystem service assessments. Int. J. Appl. Earth Obs. Geoinform..

[14] Macreadie PI (2017). Can we manage coastal ecosystems to sequester more blue carbon?. Front. Ecol. Environ..

[15] Kelleway JJ (2020). A national approach to greenhouse gas abatement through blue carbon management. Glob. Environ. Change.

[16] Luisetti T (2011). Coastal and marine ecosystem services valuation for policy and management: managed realignment case studies in England. Ocean Coast. Manag..

[17] Ellison, A. M., Felson, A. J. & Friess, D. A. Mangrove rehabilitation and restoration as experimental adaptive management. *Front. Mar. Sci*. 10.3389/fmars.2020.00327 (2020).

[18] Bertram C (2021). The blue carbon wealth of nations. Nat. Clim. Change.

[19] Bunting P (2018). The global mangrove watch–a new 2010 global baseline of mangrove extent. Remote Sens..

[20] Halpern BS (2012). An index to assess the health and benefits of the global ocean. Nature.

[21] Goldberg L, Lagomasino D, Thomas N, Fatoyinbo T (2020). Global declines in human-driven mangrove loss. Glob. Change Biol..

[22] Hagger V (2022). Drivers of global mangrove loss and gain in social-ecological systems. Nat. Commun..

[23] Duarte CM (2020). Rebuilding marine life. Nature.

[24] Friess DA (2020). Mangroves give cause for conservation optimism, for now. Curr. Biol..

[25] Gedan KB, Silliman BR, Bertness MD (2009). Centuries of human-driven change in salt marsh ecosystems. Annu. Rev. Mar. Sci..

[26] Waycott M (2009). Accelerating loss of seagrasses across the globe threatens coastal ecosystems. Proc. Natl. Acad. Sci..

[27] Krause JR, Watson EB, Wigand C, Maher N (2020). Are tidal salt marshes exposed to nutrient pollution more vulnerable to sea level rise?. Wetlands.

[28] Meera SP, Bhattacharyya M, Nizam A, Kumar A (2022). A review on microplastic pollution in the mangrove wetlands and microbial strategies for its remediation. Environ. Sci. Pollut. Res..

[29] Atwood TB (2017). Global patterns in mangrove soil carbon stocks and losses. Nat. Clim. Change.

[30] Wu J (2020). Opportunities for blue carbon strategies in China. Ocean Coast. Manag..

[31] Macreadie PI (2021). Blue carbon as a natural climate solution. Nat. Rev. Earth Environ..

[32] Jia M, Wang Z, Zhang Y, Mao D, Wang C (2018). Monitoring loss and recovery of mangrove forests during 42 years: the achievements of mangrove conservation in China. Int. J. Appl. Earth Obs. Geoinform..

[33] Hamilton SE, Casey D (2016). Creation of a high spatio-temporal resolution global database of continuous mangrove forest cover for the 21st century (CGMFC-21). Glob. Ecol. Biogeogr..

[34] Mcowen C (2017). A global map of saltmarshes. Biodivers. Data J..

[35] Friess DA, Webb EL (2014). Variability in mangrove change estimates and implications for the assessment of ecosystem service provision. Glob. Ecol. Biogeogr..

[36] Ullman R, Bilbao-Bastida V, Grimsditch G (2013). Including blue carbon in climate market mechanisms. Ocean Coast. Manag..

[37] Blue Carbon Partnership. *Protecting Mangroves, Tidal Marshes, Sea Grasses*. https://bluecarbonpartnership.org/ (2020).

[38] Menéndez P, Losada IJ, Torres-Ortega S, Narayan S, Beck MW (2020). The global flood protection benefits of mangroves. Sci. Rep..

[39] Costanza R (2021). The global value of coastal wetlands for storm protection. Glob. Environ. Change.

[40] Jänes H (2020). Quantifying fisheries enhancement from coastal vegetated ecosystems. Ecosyst. Serv..

[41] Ghermandi A, Nunes PALD (2013). A global map of coastal recreation values: results from a spatially explicit meta-analysis. Ecol. Econ..

[42] Huang B (2020). Quantifying welfare gains of coastal and estuarine ecosystem rehabilitation for recreational fisheries. Sci. Total Environ..

[43] Quevedo JMD, Uchiyama Y, Kohsaka R (2020). Perceptions of local communities on mangrove forests, their services and management: implications for eco-DRR and blue carbon management for Eastern Samar, Philippines. J. For. Res..

[44] Arumugam M, Niyomugabo R, Dahdouh-Guebas F, Hugé J (2020). The perceptions of stakeholders on current management of mangroves in the sine-Saloum delta, Senegal. Estuar. Coast. Shelf Sci..

[45] Wever L, Glaser M, Gorris P, Ferrol-Schulte D (2012). Decentralization and participation in integrated coastal management: policy lessons from Brazil and Indonesia. Ocean Coast. Manag..

[46] Merk C, Grunau J, Riekhof M-C, Rickels W (2022). The need for local governance of global commons: the example of blue carbon ecosystems. Ecol. Econ..

[47] Quevedo JMD, Uchiyama Y, Kohsaka R (2023). Progress of blue carbon research: 12 years of global trends based on content analysis of peer-reviewed and ‘gray literature’ documents. Ocean Coast. Manag..

[48] Duarte de Paula Costa M, Macreadie PI (2022). The evolution of blue carbon science. Wetlands.

[49] Page SE, Rieley JO, Banks CJ (2011). Global and regional importance of the tropical peatland carbon pool. Glob. Change Biol..

[50] Pan Y (2011). A large and persistent carbon sink in the world’s forests. Science.

[51] Gielen D (2019). The role of renewable energy in the global energy transformation. Energy Strategy Rev..

[52] Perdan S, Azapagic A (2011). Carbon trading: current schemes and future developments. Energy Policy.

[53] Bui M (2018). Carbon Capture and Storage (CCS): the way forward. Energy Environ. Sci..

[54] Abdi H, Williams LJ (2010). Principal component analysis. WIREs Comput. Stat..

[55] Matteson DS, Tsay RS (2017). Independent component analysis via distance covariance. J. Am. Stat. Assoc..

[56] OECD. *Handbook on Constructing Composite Indicators: Methodology and User Guide*. https://www.oecd.org/sdd/42495745.pdf (2008).

[57] Asbahi AA (2019). Novel approach of principal component analysis method to assess the national energy performance via energy trilemma index. Energy Rep..

[58] Blancas FJ, González M, Lozano-Oyola M, Pérez F (2010). The assessment of sustainable tourism: application to Spanish coastal destinations. Ecol. Indic..

[59] Jiang Q (2018). A principal component analysis based three-dimensional sustainability assessment model to evaluate corporate sustainable performance. J. Clean. Prod..

[60] Xu Z (2020). Assessing progress towards sustainable development over space and time. Nature.

[61] Lozano R (2018). Measuring progress from 1990 to 2017 and projecting attainment to 2030 of the health-related sustainable development goals for 195 countries and territories: a systematic analysis for the global burden of disease study 2017. Lancet.

[62] Xu Z (2020). Impacts of international trade on global sustainable development. Nat. Sustain..

[63] United Nations. *Sustainable Development Repo*rt. https://dashboards.sdgindex.org/ (2022).

[64] Zhao C, Fang C, Gong Y, Lu Z (2020). The economic feasibility of blue carbon cooperation in the South China sea region. Mar. Policy.

[65] Yang, Z. *Strategic Bargaining and Cooperation in Greenhouse Gas Mitigations: An Integrated Assessment Modeling Approach* (MIT Press, 2008).

[66] Holt CC (2004). Forecasting seasonals and trends by exponentially weighted moving averages. Int. J. Forecast..

[67] Young HP, Okada N, Hashimoto T (1982). Cost allocation in water resources development. Water Resour. Res..

[68] Shi G-M, Wang J-N, Zhang B, Zhang Z, Zhang Y-L (2016). Pollution control costs of a transboundary river basin: empirical tests of the fairness and stability of cost allocation mechanisms using game theory. J. Environ. Manage..

[69] Liu J-C (2020). Novel equal division values based on players’ excess vectors and their applications to logistics enterprise coalitions. Inf. Sci..

[70] Béal S, Casajus A, Rémila E, Solal P (2021). Cohesive efficiency in TU-games: axiomatizations of variants of the Shapley value, egalitarian values and their convex combinations. Ann. Oper. Res..

[71] Moulin H (1985). The separability axiom and equal-sharing methods. J. Econ. Theory.

[72] Ricke K, Drouet L, Caldeira K, Tavoni M (2018). Country-level social cost of carbon. Nat. Clim. Change.

[73] Friedlingstein P (2022). Global carbon budget 2022. Earth Syst. Sci. Data.

[74] Looney, B. *Statistical Review of World Energy, Energy Economics bp Global.* https://www.bp.com/en/global/corporate/energy-economics/statistical-review-of-world-energy.html (2023).

[75] Eckstein, D., Künzel, V. & Schäfer, L. *Global Climate Risk Index. Germanwatch e.V*. https://www.germanwatch.org/en/19777 (2021).

[76] Scimago Lab. *Scimago**Journal & Country Rank*. https://www.scimagojr.com/ (2022).

[77] Transparency International. *Corruption Perceptions Index.* https://www.transparency.org/en/cpi/ (2022)

[78] UNEP & IUCN. *The World Database on Protected Areas (WDPA). Protected Planet*. https://www.protectedplanet.net/en/thematic-areas/wdpa (2021).

[79] UNEP-WCMC & Short, F. T. *Global Distribution of Seagrasses.**Seventh Update to the Data Layer Used in Green and Short*. http://data.unep-wcmc.org/datasets/7 (2003)

[80] Wang F (2021). Global blue carbon accumulation in tidal wetlands increases with climate change. Natl. Sci. Rev..

[81] United Nations. *The Kyoto Protocol and Paris Agreement in United Nations Treaty Collection*. https://treaties.un.org/Pages/ViewDetails.aspx?src=TREATY&mtdsg_no=XXVII-7-a&chapter=27&clang=_en (2015).

[82] Niemeijer D, de Groot RS (2008). Framing environmental indicators: moving from causal chains to causal networks. Environ. Dev. Sustain..

[83] United Nations. *Department of Economic and Social Affairs. Indicators of Sustainable Development: Guidelines and Methodologies* 3rd edn (United Nations, 2007).

