# Supplementary material for: Sustainably developing global blue carbon for climate change mitigation and economic benefits through international cooperation
Source: Nat Commun. 2023 Oct 2;14:6144. doi: 10.1038/s41467-023-41870-x (PMC10545692; doi:10.1038/s41467-023-41870-x)
Supplement: Supplementary file 1 — Supplementary Information [file 41467_2023_41870_MOESM1_ESM.docx]

**Supplementary information for**

**Sustainably developing global blue carbon for climate change mitigation and economic benefits through international cooperation**

## Cuicui Feng1,2,3†, Guanqiong Ye1,2,3,4†*, Jiangning Zeng3, Jian Zeng5, Qutu Jiang6, Liuyue He1,2, Yaowen Zhang1, Zhenci Xu6*

1. Ocean College, Zhejiang University, Zhoushan, China
2. Donghai Laboratory, Zhoushan, China
3. Key Laboratory of Marine Ecosystem Dynamics, Second Institute of Oceanography, Ministry of Natural Resources, Hangzhou, China
4. Hainan Institute of Zhejiang University, Sanya, China
5. Zhejiang Institute of Hydraulics & Estuary, Hangzhou, China
6. Department of Geography, The University of Hong Kong, Hong Kong, China

† The first two authors contributed equally to this work

* Corresponding authors: Guanqiong Ye (gqy@zju.edu.cn) and Zhenci Xu (xuzhenci@hku.hk)

**Supplementary Note 1. Indicator selection criteria**

Our selection of indicators is based on the following principles:

1. Relevance and applicability. The indicators selected need to be relevant to the goal of the assessment objectives (blue carbon development) and applicable to countries globally;
2. Holistic and consistent with the framework. The selection of indicators is consistent with the BCDI framework that has been constructed;
3. Availability and coverage: The percentage of countries with data for an indicator is greater than 70%.

**Supplementary Note 2. Extended figures and data.**


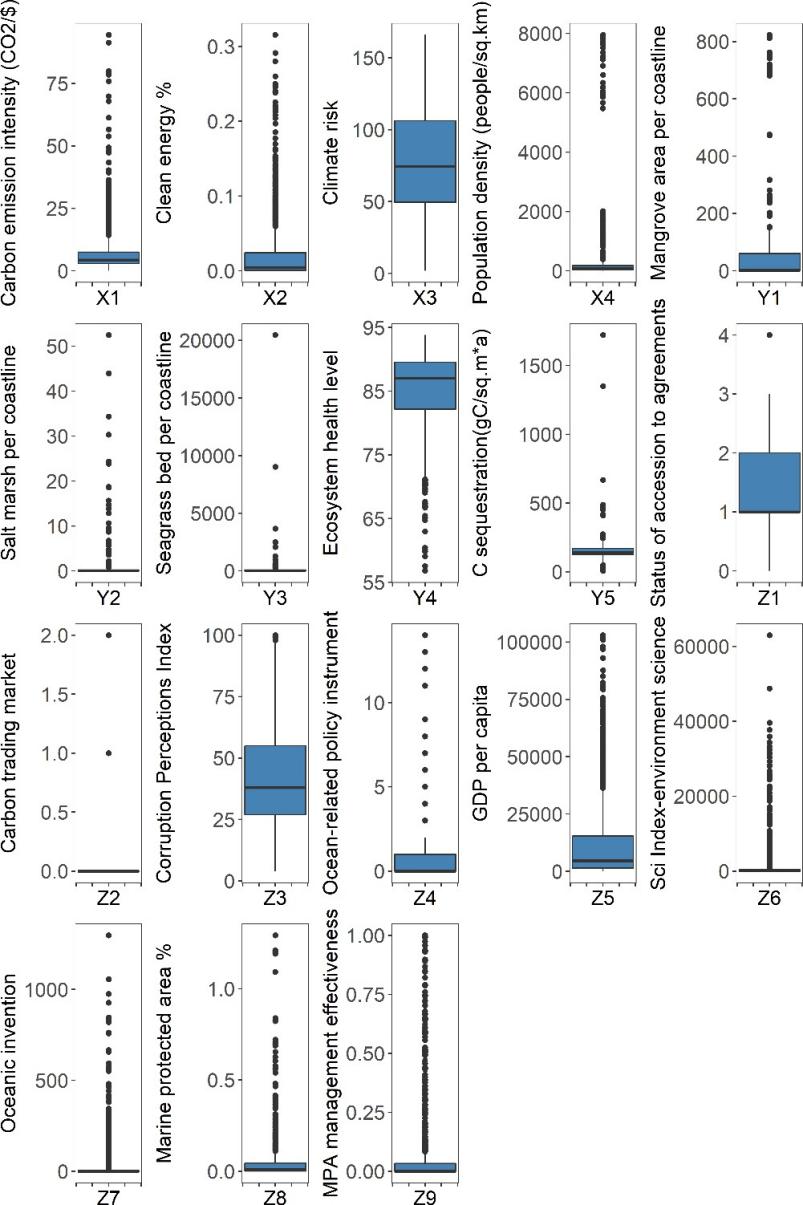


Fig. S1. Data distribution of each indicator. The data of most indicators in the development capacity and resource endowment subsystems were significantly polarized, leading to low values in most countries.The boxplot displays the median value at the centre, while the lower and upper bounds represent the first and third quartiles. The upper and lower whiskers extend from the bounds to the largest or lowest value, but no further than 1.5 times the IQR (the distance between the first and third quartiles). Sample sizes: n = 3264.


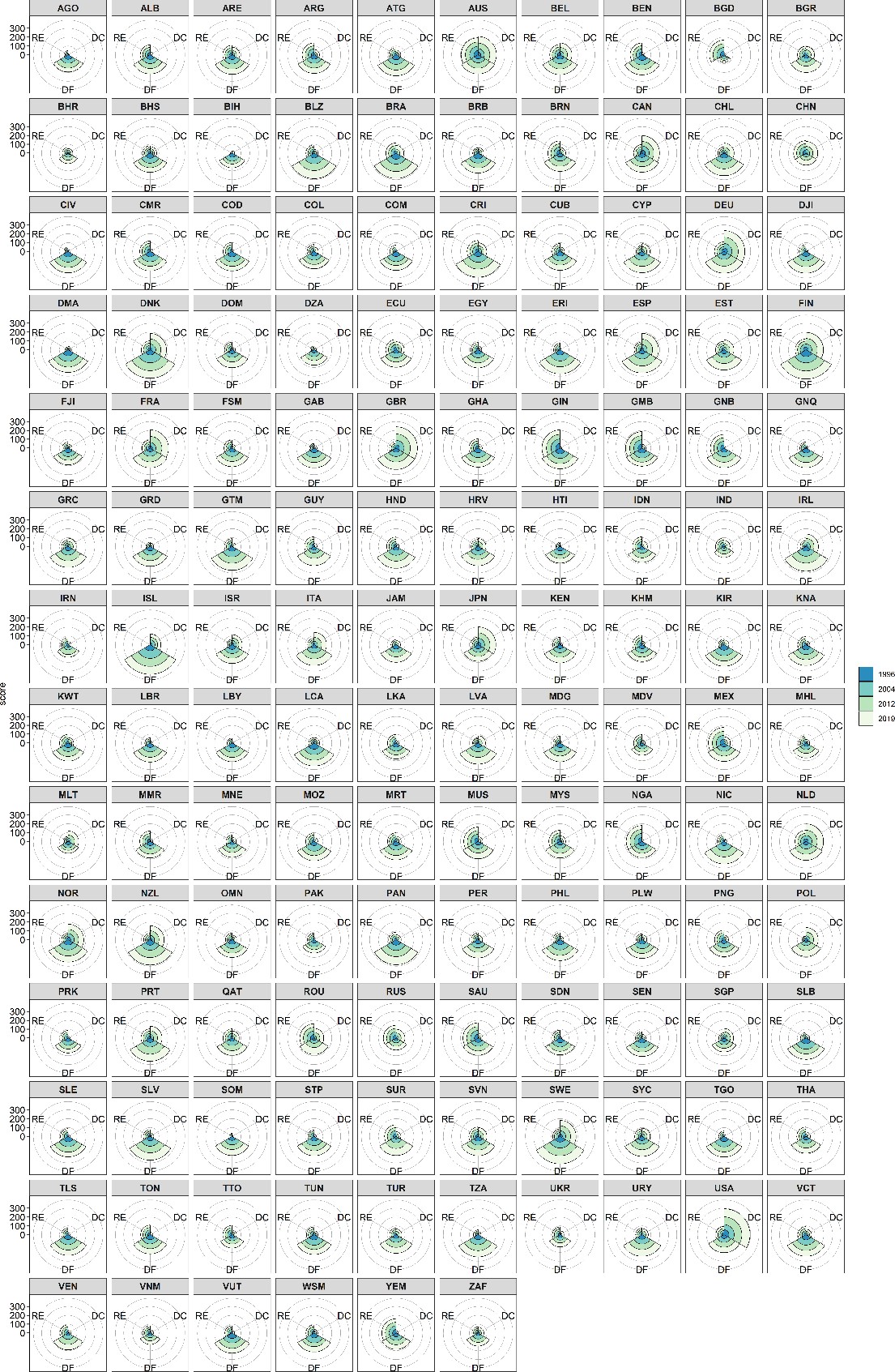


Fig. S2. Scores of three subsystems (DF-Driving Force, DC-Development Capacity, RE-Resource Endowment) of countries. The large advance in BCDI scores for countries between 1996 and 2019 was predominantly due to better performances in their development capacity and driving force subsystems. ISO3 codes are used for each country in the figure, see Table S9.


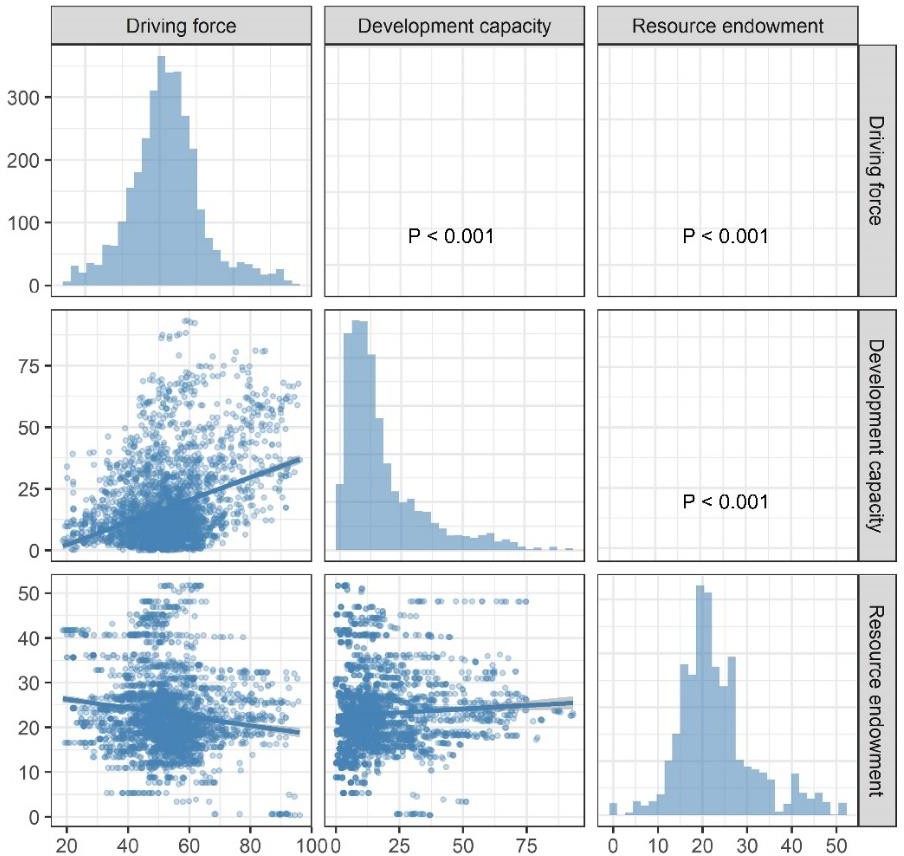


Fig.S3. Correlation analysis between three subsystems. A two-sided t-test was utilized, no adjustments were made for multiple comparisons.


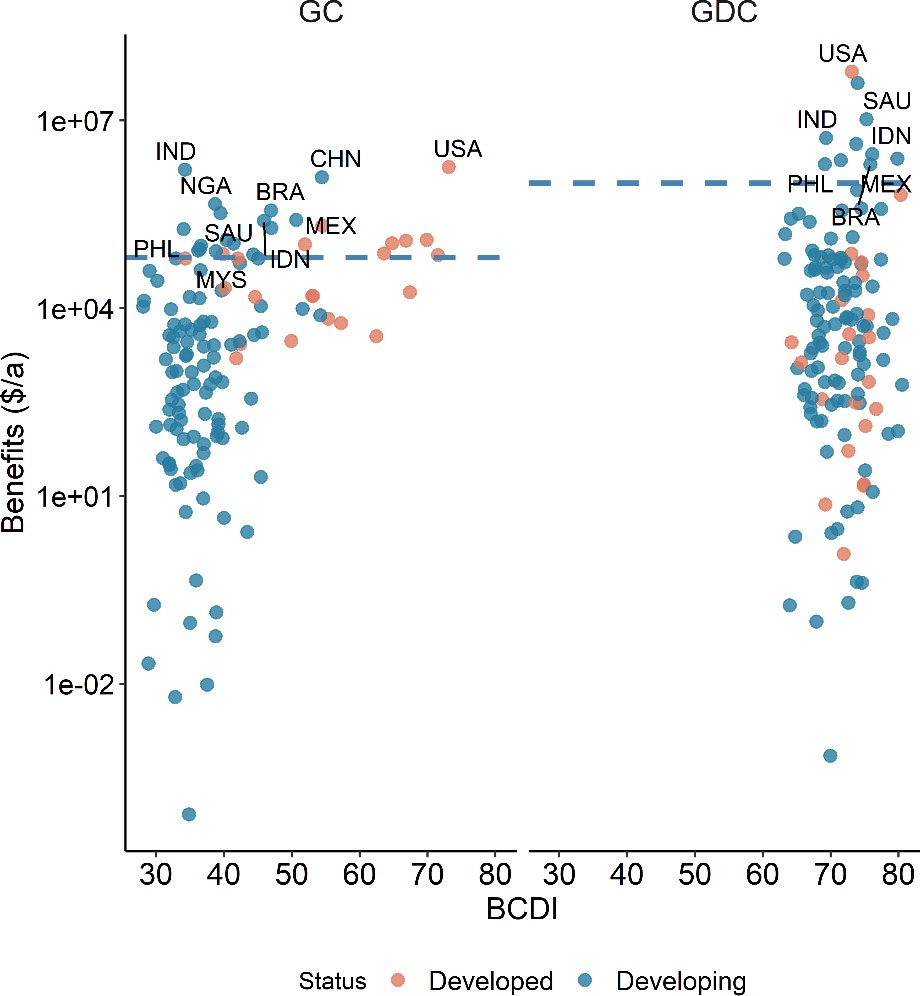


Fig. S4. Benefits of increased carbon sequestration of GC or GDC scenario compared to BAU. Blue line refers to the average value of benefit. The GDC scenario demonstrated a considerable increase in BCDI scores and economic benefits compared to the GC scenario, $136.34 million in economic benefits could be generated globally under the GDC scenario. USA-United States, SAU- Saudi Arabia, IDN- Indonesia, IND- India, NGA- Nigeria, CHN-China, MYS- Malaysia, BRA- Brazil, MEX- Mexico.

**Table. S1. Blue Carbon Development Index indicator system and the weights.**

| **Subsystems** | **Indicators** | **Property** | **Unit** | **Weight** 𝒘𝒋 |
| --- | --- | --- | --- | --- |
|  | X1-Carbon emission intensity | - | CO2 $-1 | 0.0412 |
| **Driving Force**  **(**𝒘𝒌**0.1927)** | X2-Proportion of clean energy | + | % | 0.0633 |
|  | X3-Climate change risk | + | / | 0.0452 |
|  | X4-Population density | - | people km-2 | 0.0430 |
|  | Y1-Mangrove per unit length of coastline | + | ha km-1 | 0.0639 |
| **Resource Endowment**  **(**𝒘𝒌**0.2634)** | Y2-Saltmarsh per unit length of coastline | + | ha km-1 | 0.0561 |
|  | Y3-Seagrass per unit length of coastline | + | ha km-1 | 0.0532 |
|  | Y4-Ecosystem health | + | / | 0.0543 |
|  | Y5-Rate of carbon sequestration | + | gCm-2 y-1 | 0.0359 |
|  | Z1-Status of accession to commitment agreements | + | / | 0.0559 |
|  | Z2-Construction of carbon trading market | + | / | 0.0539 |
|  | Z3-Corruption Perceptions Index | + | / | 0.0694 |
|  | Z4-Ocean-related policy instrument | + | number | 0.0501 |
| **Development Capacity**  **(**𝒘𝒌**0.5439)** | Z5-GDP per capita | + | $ per capita | 0.0690 |
|  | Z6-Sci Index of environment science | + | / | 0.0744 |
|  | Z7-Oceanic inventions | + | number | 0.0770 |
|  | Z8-Proportion of Marine Protected Area | + | % | 0.0479 |
|  | Z9-Management Effectiveness of MPA | + | % | 0.0463 |

**Table. S2. Indicators descriptions and sources**

| **Subsystems** | **Indicators** | **Units** | **Explanations** | **Indicator sources** | **Data sources** |
| --- | --- | --- | --- | --- | --- |
| Driving Force | - Carbon emission | tCO2 $^-1^ | This indicator aims to reflect the current status of carbon emission intensity and the drivers for emission reduction of  countries. | Mohsin et al., 20191 ; Xu et al., 20202 | Friedlingstein et al., 20203; World Bank4 |
|  | - Proportion   of clean energy | % | The use of clean energy sources emits less greenhouse gases than traditional fossil energy sources. This indicator is set to reflect the energy structures and energy transition drivers of each  country. | Tan et al., 20175 | Looney et al., 20206 |
|  | - Climate change risk | / | Reflects the climate change security of  countries. | Berrang-Ford et al., 20147 | Eckstein et al., 20218 |
|  | - Population density | people km-2 | This indicator reflects the population and development impact in  countries. | Tan et al., 20175 | World Bank4 |
| Development Capacity | - Status of   accession to commitme nt agreements | / | This indicator is the status of the country's signing and ratification of the Kyoto Protocol and the Paris Agreement. It reflects the country's participation in  international action against climate change. | Peters et al., 20179 | United Nations Treaty Collection, Kyoto Protocol10;  United Nations Treaty Collection, Paris Agreement11  (Assign scores according to the signing and ratification; signature: 1,  ratification: 2, maximum score of 4 for both agreements signed and  ratified) |
|  | - Constructio   n of carbon trading market | / | This indicator is the national carbon trading market situation. This indicator reflects the country's adaptation  measures to address | Perdan & Azapagic, 201112;  Jung & Song, 202313 | International Carbon Action Partnership14 (Assign points based on the construction of carbon markets. Consideration: 1  point; In force: 2 points). |

|  |  |  | climate change. |  |  |
| --- | --- | --- | --- | --- | --- |
|  | - Corruption   Perceptions Index | / | The level of corruption within an institution is one of the main factors in the governance quality. This indicator aims to reflect the comprehensive governance capacity of  countries. | Berrang-Ford et al., 20147;  Andrijevic et al., 2020 15 | Transparency International16 |
|  | - Ocean- related policy instrument | number | This indicator is intended to reflect the status and capacity of the country's management in relation  to the oceans. | Kelly et al., 201817 | OECD (Organization for Economic Co-operation and Development)18 |
|  | - GDP per   capita | $ per  capita | This indicator is intended to reflect the overall economic strength of the countries. Economic strength could to some extent influence its investment in  environmental and  marine protection. | Berrang-Ford et al., 20147 | World Bank4 |
|  | - Sci Index   of environme nt science | / | This indicator is intended to reflect the scientific and technological strength of the country's environmental protection  and restoration. | United Nations19 | SCImago20 |
|  | - Oceanic inventions | number | This indicator aims to reflect the country's innovation and scientific strength in the marine sector. | United Nations19 | OECD18  (We summarize relative inventions (1. ocean energy; 2. ocean pollution;  3. climate change mitigation in transport; 4. desalination; 5. climate change mitigation in fishing; 6. coastal adaption; 7. ocean-related envtech) from the sustainable ocean  economy database of |

|  |  |  |  |  | OECD) |
| --- | --- | --- | --- | --- | --- |
|  | - Proportion   of Marine Protected Area (MPA) | % | With human  development and ocean exploitation, marine ecosystems are facing serious problems such as degradation and  pollution. The establishment of marine protected areas helps to maintain the fragile marine ecosystems and marine biodiversity. This indicator reflects the current status and concerns of marine  conservation in countries. | Mascia et al., 201721 | UNEP-WCMC and IUCN, 202122  (Aggregate the MPA area of each country, and then divide by the territorial water area) |
|  | - Manageme   nt Effectivene ss of MPA | % | Management effectiveness refers to how well protected areas are managed - primarily the extent to which management protects values and achieves goals and targets. This indicator is intended to reflect the level of management of the country's marine  conservation. | Fox et al., 201423; Gill et al., 201724 | UNEP-WCMC and IUCN, 202122  (The overall management effectiveness of the country = (area of MPAs for which management assessments were conducted) / (total area of MPAs)) |
| Resource Endowment | - Mangrove   area per unit length of coastline | ha per km coastline | Mangroves are one of the blue carbon ecosystems, mainly distributed in tropical and subtropical regions. This indicator reflects the resources of blue carbon ecosystems. | Richards et al., (2020) 25 | Bunting et al., 201826 (Obtain the geographical information layer, perform Mollweide Projection, then calculate the patch area through ArcGIS, and aggregate the mangrove area of each country, and then divide by the coastline  length27) |
|  | - Saltmarsh   area per unit length of coastline | ha per km coastline | Salt marshes are one of the blue carbon ecosystems, which are  widely distributed in | Cisneros- Montemayor et al., 202128 | Mcowen et al., 201729 (Obtain the geographical information layer of global  salt marsh distribution |

|  |  |  | coastal zone areas around the world, especially in estuaries. This indicator reflects the resources of blue carbon ecosystems. |  | from UNEP 2020, perform Mollweide Projection, then calculate the patch area through ArcGIS, and aggregate the mangrove area of each country, and then divide by the coastline  length) |
| --- | --- | --- | --- | --- | --- |
|  | - Seagrass area per unit length of coastline | ha per km coastline | Seagrass bed ecosystem is one of the blue carbon ecosystems, which is widely distributed in shallow marine areas around the world. This indicator reflects the resources of blue carbon  ecosystems. | Cisneros- Montemayor et al., 202128 | UNEP 202030  (Obtain the geographical information layer, perform Mollweide Projection, then calculate the patch area through ArcGIS, and aggregate the seagrass area of each country, and then  divide by coastline length) |
|  | - Ecosystem health | / | Reflects the level of health of marine ecosystems in each  country. | Halpern et al., 201231 | Halpern et al., 201231 |
|  | - Rate of   carbon sequestrati on | gC m-2 y-1 | The indicator reflects the overall efficient of carbon sink functions of the blue carbon ecosystem in countries. | Ouyang & Lee, 2020  32 | Bunting et al., 201826; Mcowen et al., 201729; UNEP 202030;  Wang et al., 202133; Mcleod et al., 201134 (First, calculate annual carbon burial based on geographic data of blue carbon (indicators 14, 15, and 16) and carbon sequestration rate33; Mcleod et al., 201134), and then calculate the country- specific average carbon  sequestration rate.) |

**Table. S3. Scores of BCDI, driving force, development capacity, and resource endowment of regions.**

|  |  | 1996 | 1997 | 1998 | 1999 | 2000 | 2001 | 2002 | 2003 | 2004 | 2005 | 2006 | 2007 | 2008 | 2009 | 2010 | 2011 | 2012 | 2013 | 2014 | 2015 | 2016 | 2017 | 2018 | 2019 |
| --- | --- | --- | --- | --- | --- | --- | --- | --- | --- | --- | --- | --- | --- | --- | --- | --- | --- | --- | --- | --- | --- | --- | --- | --- | --- |
| BCDI | Central and Southern Asia | 14.49 | 14.64 | 14.99 | 14.88 | 14.98 | 15.28 | 15.77 | 16.02 | 16.00 | 16.42 | 16.77 | 17.29 | 17.76 | 18.20 | 18.72 | 18.61 | 19.45 | 19.55 | 19.53 | 19.74 | 22.36 | 22.42 | 23.28 | 22.56 |
|  | Eastern and South-Eastern Asia | 18.93 | 18.75 | 18.94 | 19.56 | 19.61 | 19.71 | 20.54 | 21.09 | 21.39 | 21.75 | 22.50 | 23.72 | 23.84 | 24.02 | 24.89 | 24.85 | 26.75 | 26.43 | 26.82 | 27.16 | 29.67 | 30.50 | 31.15 | 31.48 |
|  | Europe and Northern America | 25.27 | 25.33 | 26.61 | 26.96 | 27.04 | 27.29 | 28.88 | 30.05 | 30.29 | 34.75 | 35.77 | 37.62 | 38.50 | 38.99 | 39.61 | 40.14 | 40.67 | 41.62 | 42.01 | 41.88 | 44.67 | 45.12 | 45.74 | 46.24 |
|  | Latin America and the  Caribbean | 19.48 | 19.50 | 20.08 | 20.84 | 21.26 | 21.33 | 21.61 | 21.65 | 21.80 | 22.49 | 23.04 | 23.05 | 22.84 | 23.71 | 23.99 | 23.94 | 25.51 | 25.44 | 26.09 | 26.68 | 28.68 | 29.38 | 30.80 | 31.11 |
|  | Northern Africa and Western  Asia | 19.17 | 19.28 | 19.10 | 19.49 | 19.86 | 19.98 | 19.81 | 20.29 | 20.28 | 21.84 | 22.38 | 22.46 | 23.12 | 22.38 | 23.13 | 23.79 | 24.73 | 24.38 | 24.97 | 24.48 | 25.71 | 27.13 | 27.13 | 27.82 |
|  | Oceania | 20.56 | 20.57 | 21.37 | 21.77 | 21.91 | 22.04 | 22.33 | 22.70 | 23.06 | 23.40 | 24.38 | 24.66 | 25.19 | 25.44 | 26.10 | 26.38 | 26.81 | 27.33 | 27.40 | 27.83 | 31.15 | 31.38 | 31.03 | 31.65 |
|  | Sub-Saharan Africa | 18.13 | 18.11 | 18.18 | 18.08 | 18.22 | 18.60 | 19.05 | 19.36 | 19.05 | 19.63 | 20.06 | 20.18 | 20.46 | 20.70 | 21.03 | 21.15 | 22.01 | 21.69 | 22.04 | 22.14 | 24.42 | 24.66 | 25.01 | 25.28 |
|  | Global | 20.26 | 20.28 | 20.79 | 21.15 | 21.35 | 21.55 | 22.18 | 22.66 | 22.74 | 24.25 | 24.93 | 25.54 | 25.90 | 26.23 | 26.75 | 26.97 | 27.99 | 28.09 | 28.51 | 28.66 | 31.00 | 31.57 | 32.18 | 32.55 |
| Driving force | Central and Southern Asia | 32.00 | 31.75 | 31.42 | 30.94 | 31.32 | 31.24 | 30.84 | 31.66 | 31.95 | 30.93 | 31.49 | 33.09 | 34.56 | 36.46 | 37.48 | 35.08 | 37.80 | 36.62 | 35.27 | 36.96 | 36.11 | 35.58 | 39.47 | 35.13 |
|  | Eastern and South-Eastern Asia | 42.46 | 41.81 | 40.65 | 41.97 | 42.26 | 41.44 | 41.73 | 42.12 | 40.12 | 39.22 | 40.28 | 45.50 | 44.33 | 44.42 | 46.41 | 44.61 | 50.34 | 46.46 | 47.96 | 49.42 | 48.91 | 49.62 | 50.48 | 51.05 |
|  | Europe and Northern America | 47.27 | 47.38 | 48.05 | 48.78 | 48.98 | 49.23 | 50.20 | 51.49 | 49.62 | 50.22 | 51.25 | 55.07 | 56.33 | 57.48 | 58.82 | 59.73 | 61.68 | 63.68 | 64.91 | 67.07 | 67.66 | 67.08 | 68.21 | 69.73 |
|  | Latin America and the  Caribbean | 52.03 | 52.17 | 51.33 | 51.94 | 52.12 | 51.90 | 51.88 | 51.63 | 51.55 | 53.84 | 54.01 | 53.10 | 50.97 | 54.07 | 54.92 | 54.36 | 59.91 | 59.19 | 60.93 | 63.44 | 61.57 | 62.31 | 67.36 | 68.43 |
|  | Northern Africa and Western  Asia | 47.17 | 47.55 | 46.79 | 47.35 | 48.28 | 47.98 | 47.51 | 48.28 | 45.42 | 45.85 | 46.52 | 47.42 | 47.73 | 45.19 | 47.74 | 48.97 | 52.98 | 49.19 | 52.72 | 51.11 | 48.37 | 52.66 | 51.18 | 53.45 |
|  | Oceania | 53.44 | 53.28 | 52.95 | 53.03 | 52.78 | 52.31 | 52.28 | 52.57 | 50.63 | 52.17 | 52.55 | 50.17 | 51.53 | 52.22 | 54.04 | 55.16 | 54.99 | 57.12 | 53.93 | 54.86 | 57.42 | 58.66 | 55.84 | 59.07 |
|  | Sub-Saharan Africa | 53.64 | 53.65 | 53.80 | 53.53 | 53.69 | 52.95 | 53.13 | 53.85 | 51.57 | 51.10 | 52.34 | 51.63 | 52.33 | 52.43 | 52.87 | 53.08 | 55.32 | 54.01 | 55.96 | 56.58 | 56.36 | 56.25 | 56.82 | 57.74 |
|  | Global | 49.11 | 49.13 | 48.88 | 49.29 | 49.54 | 49.23 | 49.43 | 50.00 | 48.41 | 49.02 | 49.79 | 50.73 | 50.77 | 51.63 | 52.89 | 52.97 | 56.23 | 55.58 | 56.85 | 58.15 | 57.65 | 58.29 | 59.65 | 60.83 |
| Development capacity | Central and Southern Asia | 2.84 | 3.21 | 3.97 | 3.93 | 3.98 | 4.56 | 5.62 | 5.80 | 5.65 | 6.78 | 7.23 | 7.62 | 7.96 | 8.10 | 8.70 | 9.35 | 9.92 | 10.62 | 11.18 | 11.01 | 16.13 | 16.42 | 16.65 | 16.85 |
|  | Eastern and South-Eastern Asia | 7.42 | 7.31 | 8.08 | 8.74 | 8.73 | 9.20 | 10.67 | 11.55 | 12.81 | 13.80 | 14.79 | 15.19 | 15.83 | 16.12 | 17.02 | 17.59 | 19.05 | 19.99 | 19.98 | 20.18 | 24.88 | 26.25 | 27.21 | 27.63 |
|  | Europe and Northern America | 18.90 | 18.98 | 21.09 | 21.48 | 21.56 | 21.92 | 24.51 | 26.20 | 27.31 | 35.29 | 36.79 | 38.84 | 40.03 | 40.52 | 41.18 | 41.84 | 42.12 | 43.13 | 43.55 | 42.67 | 47.69 | 48.90 | 49.77 | 50.16 |
|  | Latin America and the  Caribbean | 6.52 | 6.53 | 7.87 | 9.06 | 9.76 | 9.97 | 10.52 | 10.68 | 10.97 | 11.43 | 12.38 | 12.73 | 13.10 | 13.59 | 13.84 | 13.94 | 14.85 | 15.03 | 15.53 | 15.70 | 20.10 | 21.11 | 21.92 | 22.10 |
|  | Northern Africa and Western  Asia | 7.56 | 7.62 | 7.56 | 8.08 | 8.44 | 8.76 | 8.61 | 9.21 | 10.22 | 12.93 | 13.69 | 13.52 | 14.62 | 14.15 | 14.64 | 15.42 | 15.72 | 16.54 | 16.32 | 16.08 | 19.40 | 20.47 | 21.12 | 21.58 |
|  | Oceania | 7.80 | 7.89 | 9.46 | 10.18 | 10.52 | 10.93 | 11.48 | 12.05 | 13.41 | 13.47 | 15.15 | 16.51 | 16.99 | 17.21 | 17.77 | 17.91 | 18.74 | 19.00 | 20.19 | 20.68 | 25.89 | 26.00 | 26.36 | 26.36 |
|  | Sub-Saharan Africa | 2.73 | 2.70 | 2.77 | 2.69 | 2.89 | 3.84 | 4.64 | 4.95 | 5.19 | 6.41 | 6.76 | 7.24 | 7.51 | 7.92 | 8.36 | 8.50 | 9.30 | 9.29 | 9.36 | 9.40 | 13.77 | 14.20 | 14.71 | 14.87 |
|  | Global | 8.59 | 8.63 | 9.65 | 10.18 | 10.46 | 10.94 | 12.02 | 12.71 | 13.41 | 15.99 | 16.96 | 17.75 | 18.39 | 18.70 | 19.21 | 19.59 | 20.30 | 20.80 | 21.13 | 21.01 | 25.55 | 26.41 | 27.10 | 27.36 |

| Resource endowment | Central and Southern Asia | 25.73 | 25.73 | 25.73 | 25.73 | 25.73 | 25.73 | 25.70 | 25.70 | 25.70 | 25.70 | 25.70 | 25.70 | 25.70 | 25.69 | 25.70 | 25.70 | 25.70 | 25.49 | 25.25 | 25.16 | 25.18 | 25.19 | 25.14 | 25.14 |
| --- | --- | --- | --- | --- | --- | --- | --- | --- | --- | --- | --- | --- | --- | --- | --- | --- | --- | --- | --- | --- | --- | --- | --- | --- | --- |
|  | Eastern and South-Eastern Asia | 25.50 | 25.50 | 25.50 | 25.50 | 25.50 | 25.50 | 25.40 | 25.40 | 25.40 | 25.40 | 25.40 | 25.40 | 25.40 | 25.40 | 25.39 | 25.39 | 25.39 | 25.09 | 25.50 | 25.29 | 25.47 | 25.26 | 25.12 | 25.12 |
|  | Europe and Northern America | 22.32 | 22.32 | 22.32 | 22.32 | 22.32 | 22.32 | 22.32 | 22.32 | 22.32 | 22.32 | 22.32 | 22.32 | 22.32 | 22.32 | 22.32 | 22.32 | 22.32 | 22.36 | 22.07 | 21.80 | 21.62 | 21.26 | 20.99 | 20.99 |
|  | Latin America and the  Caribbean | 22.41 | 22.41 | 22.41 | 22.41 | 22.41 | 22.41 | 22.39 | 22.39 | 22.39 | 22.39 | 22.39 | 22.39 | 22.39 | 22.38 | 22.34 | 22.34 | 22.34 | 22.24 | 22.42 | 22.46 | 22.32 | 22.36 | 22.40 | 22.40 |
|  | Northern Africa and Western  Asia | 22.67 | 22.67 | 22.67 | 22.67 | 22.67 | 22.67 | 22.67 | 22.67 | 22.67 | 22.67 | 22.67 | 22.67 | 22.67 | 22.67 | 22.67 | 22.67 | 22.67 | 22.43 | 22.52 | 22.34 | 22.14 | 22.20 | 21.96 | 21.96 |
|  | Oceania | 22.84 | 22.84 | 22.84 | 22.84 | 22.84 | 22.84 | 22.84 | 22.84 | 22.84 | 22.84 | 22.84 | 22.84 | 22.84 | 22.84 | 22.84 | 22.84 | 22.84 | 22.74 | 22.86 | 22.84 | 22.79 | 22.52 | 22.53 | 22.53 |
|  | Sub-Saharan Africa | 23.94 | 23.94 | 23.94 | 23.94 | 23.94 | 23.94 | 23.89 | 23.89 | 23.89 | 23.89 | 23.89 | 23.89 | 23.89 | 23.88 | 23.89 | 23.89 | 23.89 | 23.64 | 23.41 | 23.26 | 23.03 | 23.13 | 23.02 | 23.02 |
|  | Global | 23.23 | 23.23 | 23.23 | 23.23 | 23.23 | 23.23 | 23.20 | 23.20 | 23.20 | 23.20 | 23.20 | 23.20 | 23.20 | 23.19 | 23.19 | 23.19 | 23.19 | 23.05 | 23.02 | 22.89 | 22.77 | 22.68 | 22.57 | 22.57 |

**Table. S4. Carbon sequestration increase allocation in scenarios at national scale (t C/a)**

| **Region** | **Status** | **ISO3** | **BAU** | **GC allocation** | **GDC allocation** |
| --- | --- | --- | --- | --- | --- |
| Sub-Saharan Africa | Developing | AGO | 3759.69 | 4597.69 | 8631.73 |
| Europe and Northern America | Developing | ALB | 147.20 | 169.63 | 379.56 |
| Northern Africa and Western Asia | Developing | ARE | 1053.56 | 5566.60 | 2262.39 |
| Latin America and the Caribbean | Developing | ARG | 612.55 | 5125.58 | 937.66 |
| Latin America and the Caribbean | Developing | ATG | 295.36 | 327.61 | 814.68 |
| Oceania | Developed | AUS | 56147.55 | 60660.59 | 79826.28 |
| Europe and Northern America | Developed | BEL | 21.66 | 4534.70 | 26.13 |
| Sub-Saharan Africa | Developing | BEN | 6211.54 | 7259.63 | 14338.38 |
| Central and Southern Asia | Developing | BGD | 2200.42 | 2534.57 | 4879.08 |
| Europe and Northern America | Developing | BGR | 1.30 | 4514.34 | 1.75 |
| Northern Africa and Western Asia | Developing | BHR | 322.11 | 390.64 | 1122.75 |
| Latin America and the Caribbean | Developing | BHS | -9614.59 | -9441.23 | -3665.92 |
| Europe and Northern America | Developing | BIH | 0.39 | 0.49 | 1.29 |
| Latin America and the Caribbean | Developing | BLZ | 2496.71 | 3439.82 | 13864.14 |
| Latin America and the Caribbean | Developing | BRA | -28255.58 | -23742.54 | -3657.61 |
| Latin America and the Caribbean | Developing | BRB | 179.24 | 191.00 | 361.17 |
| Eastern and South-Eastern Asia | Developing | BRN | -2005.70 | -1904.49 | 840.35 |
| Europe and Northern America | Developed | CAN | 11614.32 | 16127.35 | 12855.18 |
| Latin America and the Caribbean | Developing | CHL | 12.70 | 4525.73 | 15.29 |
| Eastern and South-Eastern Asia | Developing | CHN | 16504.96 | 21018.00 | 27038.47 |
| Sub-Saharan Africa | Developing | CIV | -932.41 | -877.17 | -492.73 |
| Sub-Saharan Africa | Developing | CMR | 2769.03 | 2990.50 | 5227.91 |
| Sub-Saharan Africa | Developing | COD | -148.74 | -100.90 | 235.94 |
| Latin America and the Caribbean | Developing | COL | -37508.88 | -32995.85 | -25211.05 |
| Sub-Saharan Africa | Developing | COM | 4488.58 | 5043.79 | 8271.32 |
| Latin America and the Caribbean | Developing | CRI | -3486.14 | 1026.90 | -1797.30 |
| Latin America and the Caribbean | Developing | CUB | 1263.01 | 4868.15 | 41466.94 |
| Northern Africa and Western Asia | Developed | CYP | 222.79 | 4735.82 | 312.20 |
| Europe and Northern America | Developed | DEU | 6410.47 | 10923.51 | 6882.36 |
| Sub-Saharan Africa | Developing | DJI | 0.74 | 1.01 | 2.72 |
| Latin America and the Caribbean | Developing | DMA | 1270.19 | 1380.23 | 2457.23 |
| Europe and Northern America | Developed | DNK | 8179.20 | 12692.24 | 9016.79 |
| Latin America and the Caribbean | Developing | DOM | 318.00 | 460.20 | 1861.23 |
| Northern Africa and Western Asia | Developing | DZA | 44.14 | 56.03 | 145.93 |
| Latin America and the Caribbean | Developing | ECU | -16818.90 | -12305.86 | -10715.01 |
| Northern Africa and Western Asia | Developing | EGY | 4564.21 | 4759.82 | 8949.02 |
| Sub-Saharan Africa | Developing | ERI | -0.25 | 7.78 | 45.81 |
| Europe and Northern America | Developed | ESP | 16761.82 | 21274.86 | 18140.57 |
| Europe and Northern America | Developed | EST | 18.34 | 4531.38 | 22.46 |
| Europe and Northern America | Developed | FIN | 768.58 | 5281.62 | 824.35 |
| Oceania | Developing | FJI | 1447.30 | 1880.84 | 5830.25 |

| Europe and Northern America | Developed | FRA | 13982.00 | 18495.03 | 16009.26 |
| --- | --- | --- | --- | --- | --- |
| Oceania | Developing | FSM | 296.25 | 340.42 | 696.59 |
| Sub-Saharan Africa | Developing | GAB | 970.23 | 1131.32 | 3100.64 |
| Europe and Northern America | Developed | GBR | 203.38 | 4716.41 | 423.48 |
| Sub-Saharan Africa | Developing | GHA | 11424.13 | 11871.36 | 22301.99 |
| Sub-Saharan Africa | Developing | GIN | 217749.02 | 244779.79 | 417883.16 |
| Sub-Saharan Africa | Developing | GMB | 5196.04 | 5433.56 | 8796.28 |
| Sub-Saharan Africa | Developing | GNB | 22053.62 | 24734.71 | 43066.26 |
| Sub-Saharan Africa | Developing | GNQ | 90.45 | 137.59 | 474.18 |
| Europe and Northern America | Developed | GRC | 1955.99 | 6469.02 | 2786.98 |
| Latin America and the Caribbean | Developing | GRD | 19.97 | 22.16 | 43.89 |
| Latin America and the Caribbean | Developing | GTM | -175.52 | -143.87 | 198.12 |
| Latin America and the Caribbean | Developing | GUY | -297.54 | -274.39 | 562.69 |
| Latin America and the Caribbean | Developing | HND | -3608.73 | -2422.70 | 6450.17 |
| Europe and Northern America | Developing | HRV | 1000.43 | 5513.46 | 1370.95 |
| Latin America and the Caribbean | Developing | HTI | 642.06 | 1016.25 | 3098.03 |
| Eastern and South-Eastern Asia | Developing | IDN | -93274.26 | -88761.22 | 95755.71 |
| Central and Southern Asia | Developing | IND | 2326.75 | 6839.78 | 16891.27 |
| Europe and Northern America | Developed | IRL | 96.29 | 4609.32 | 118.25 |
| Central and Southern Asia | Developing | IRN | 6414.67 | 6971.74 | 12824.76 |
| Europe and Northern America | Developed | ISL | 14.64 | 4527.67 | 19.94 |
| Northern Africa and Western Asia | Developed | ISR | 63.36 | 4576.40 | 85.01 |
| Europe and Northern America | Developed | ITA | 17662.56 | 22175.60 | 20835.35 |
| Latin America and the Caribbean | Developing | JAM | 772.53 | 836.83 | 2384.62 |
| Eastern and South-Eastern Asia | Developed | JPN | 610.55 | 5123.58 | 906.12 |
| Sub-Saharan Africa | Developing | KEN | -442.00 | -335.83 | 658.69 |
| Eastern and South-Eastern Asia | Developing | KHM | -6217.92 | -6005.62 | -4082.84 |
| Oceania | Developing | KIR | 762.40 | 5275.44 | 1220.93 |
| Latin America and the Caribbean | Developing | KNA | 89.53 | 99.47 | 217.86 |
| Northern Africa and Western Asia | Developing | KWT | 3251.75 | 3473.33 | 6615.86 |
| Sub-Saharan Africa | Developing | LBR | 382.71 | 523.01 | 1721.80 |
| Northern Africa and Western Asia | Developing | LBY | 1.85 | 7.23 | 36.93 |
| Latin America and the Caribbean | Developing | LCA | 5.60 | 7.29 | 26.34 |
| Central and Southern Asia | Developing | LKA | 3802.77 | 4773.57 | 11804.25 |
| Europe and Northern America | Developed | LVA | 2.95 | 4515.99 | 3.96 |
| Sub-Saharan Africa | Developing | MDG | 22336.97 | 24387.47 | 40499.73 |
| Central and Southern Asia | Developing | MDV | 2981.03 | 3674.15 | 9002.75 |
| Latin America and the Caribbean | Developing | MEX | -15889.35 | -11376.31 | 26935.79 |
| Oceania | Developing | MHL | 744.19 | 875.89 | 2000.99 |
| Europe and Northern America | Developed | MLT | 439.59 | 4952.63 | 537.84 |
| Eastern and South-Eastern Asia | Developing | MMR | -6342.83 | -5202.80 | 8884.94 |
| Europe and Northern America | Developing | MNE | 2.47 | 2.63 | 5.05 |
| Sub-Saharan Africa | Developing | MOZ | -10076.95 | -9715.28 | -4485.54 |

| Sub-Saharan Africa | Developing | MRT | -9.92 | -9.84 | -7.81 |
| --- | --- | --- | --- | --- | --- |
| Sub-Saharan Africa | Developing | MUS | 9524.06 | 10190.25 | 22040.98 |
| Eastern and South-Eastern Asia | Developing | MYS | -26780.60 | -26195.00 | 66743.84 |
| Sub-Saharan Africa | Developing | NGA | 73896.73 | 79709.70 | 127086.05 |
| Latin America and the Caribbean | Developing | NIC | 1623.37 | 4459.11 | 22733.69 |
| Europe and Northern America | Developed | NLD | 150.26 | 4663.30 | 212.02 |
| Europe and Northern America | Developed | NOR | 265.99 | 4779.02 | 305.78 |
| Oceania | Developed | NZL | -945.26 | 3567.78 | -739.56 |
| Northern Africa and Western Asia | Developing | OMN | -0.89 | -0.86 | -0.56 |
| Central and Southern Asia | Developing | PAK | -6861.74 | -6294.41 | -187.99 |
| Latin America and the Caribbean | Developing | PAN | 2090.95 | 3033.50 | 21118.39 |
| Latin America and the Caribbean | Developing | PER | 540.76 | 565.06 | 1355.13 |
| Eastern and South-Eastern Asia | Developing | PHL | 22149.97 | 29339.05 | 100730.97 |
| Oceania | Developing | PLW | 1463.63 | 1484.40 | 2750.19 |
| Oceania | Developing | PNG | 36168.17 | 39061.20 | 84450.49 |
| Europe and Northern America | Developed | POL | 10.51 | 4523.55 | 12.70 |
| Eastern and South-Eastern Asia | Developing | PRK | 6.10 | 8.49 | 25.00 |
| Europe and Northern America | Developed | PRT | 189.10 | 4702.14 | 227.16 |
| Northern Africa and Western Asia | Developing | QAT | 1379.83 | 5892.87 | 3438.57 |
| Europe and Northern America | Developing | ROU | 250.60 | 4763.64 | 314.89 |
| Europe and Northern America | Developing | RUS | 32876.68 | 37389.71 | 43958.55 |
| Northern Africa and Western Asia | Developing | SAU | 461888.87 | 465156.81 | 854498.89 |
| Northern Africa and Western Asia | Developing | SDN | 13215.63 | 15162.73 | 26457.60 |
| Sub-Saharan Africa | Developing | SEN | 4352.68 | 5106.49 | 17421.77 |
| Eastern and South-Eastern Asia | Developed | SGP | 138.68 | 4651.71 | 345.37 |
| Oceania | Developing | SLB | 2356.46 | 2587.54 | 5524.24 |
| Sub-Saharan Africa | Developing | SLE | 12306.64 | 13290.34 | 21474.44 |
| Latin America and the Caribbean | Developing | SLV | 31.08 | 104.97 | 745.28 |
| Sub-Saharan Africa | Developing | SOM | 22.40 | 26.50 | 49.34 |
| Sub-Saharan Africa | Developing | STP | 0.83 | 0.83 | 0.84 |
| Latin America and the Caribbean | Developing | SUR | 9105.26 | 9192.75 | 11693.16 |
| Europe and Northern America | Developed | SVN | 9.30 | 4522.33 | 12.69 |
| Europe and Northern America | Developed | SWE | 464.04 | 4977.08 | 519.19 |
| Sub-Saharan Africa | Developing | SYC | 3.45 | 4516.49 | 4.29 |
| Sub-Saharan Africa | Developing | TGO | 216.04 | 353.13 | 1209.65 |
| Eastern and South-Eastern Asia | Developing | THA | 736.40 | 5249.44 | 15221.16 |
| Eastern and South-Eastern Asia | Developing | TLS | -1.40 | 5.00 | 48.91 |
| Oceania | Developing | TON | 6899.06 | 7990.80 | 16249.52 |
| Latin America and the Caribbean | Developing | TTO | -740.00 | -719.15 | -523.84 |
| Northern Africa and Western Asia | Developing | TUN | 7512.96 | 8551.49 | 18755.52 |
| Northern Africa and Western Asia | Developing | TUR | 32.33 | 4545.37 | 50.21 |
| Sub-Saharan Africa | Developing | TZA | 148.12 | 257.40 | 1593.79 |
| Europe and Northern America | Developing | UKR | 25703.41 | 26747.36 | 48144.82 |

| Latin America and the Caribbean | Developing | URY | 11.52 | 11.69 | 19.56 |
| --- | --- | --- | --- | --- | --- |
| Europe and Northern America | Developed | USA | -9372.61 | -4859.57 | 141509.08 |
| Latin America and the Caribbean | Developing | VCT | 3.75 | 4.09 | 10.87 |
| Latin America and the Caribbean | Developing | VEN | 3067.00 | 5061.91 | 21120.05 |
| Eastern and South-Eastern Asia | Developing | VNM | -11928.34 | -11690.71 | -7005.11 |
| Oceania | Developing | VUT | 3128.13 | 3426.67 | 6069.68 |
| Oceania | Developing | WSM | 1049.56 | 1311.51 | 3450.12 |
| Northern Africa and Western Asia | Developing | YEM | 137760.53 | 149752.50 | 251370.74 |
| Sub-Saharan Africa | Developing | ZAF | -0.77 | 4512.27 | 26.83 |

**Table. S5. Benefits of increased carbon sequestration of GC or GDC scenario ($/a)**

| **ISO3** | **GC benefits** | **GDC benefits** | **ISO3** | **GC benefits** | **GDC benefits** |
| --- | --- | --- | --- | --- | --- |
| AGO | 10613.77 | 61707.60 | KIR | 62691.62 | 6369.52 |
| ALB | 9.14 | 94.63 | KNA | 138.11 | 1782.67 |
| ARE | 190254.06 | 50960.08 | KWT | 4270.66 | 64837.98 |
| ARG | 71813.67 | 5173.37 | LBR | 118.30 | 1129.06 |
| ATG | 447.94 | 7213.96 | LBY | 31.46 | 205.01 |
| AUS | 123567.99 | 648329.08 | LCA | 23.35 | 288.07 |
| BEL | 15781.94 | 15.61 | LKA | 5452.58 | 44940.83 |
| BEN | 2442.17 | 18936.36 | LVA | -24.88 | -0.01 |
| BGD | 5525.49 | 44294.57 | MDG | 6093.86 | 53977.52 |
| BGR | 4112.21 | 0.41 | MDV | 9628.25 | 83648.99 |
| BHR | 951.87 | 11121.80 | MEX | 257729.62 | 2445649.77 |
| BHS | 67.55 | 2317.98 | MHL | 1829.46 | 17458.49 |
| BIH | 0.02 | 0.18 | MLT | 62691.62 | 1364.76 |
| BLZ | 124.05 | 1495.22 | MMR | 4536.09 | 60590.28 |
| BRA | 365501.13 | 1992136.45 | MNE | 0.01 | 0.10 |
| BRB | 163.35 | 2527.22 | MOZ | 1641.60 | 25379.03 |
| BRN | 105.56 | 2968.42 | MRT | 0.09 | 2.57 |
| CAN | 5771.87 | 1586.98 | MUS | 360.34 | 6770.33 |
| CHL | 9825.07 | 5.64 | MYS | 14493.16 | 2314646.96 |
| CHN | 1239911.82 | 2893976.33 | NGA | 461743.40 | 4225004.00 |
| CIV | 240.68 | 1915.77 | NIC | 2572.59 | 19151.32 |
| CMR | 952.01 | 10569.79 | NLD | 18007.15 | 246.42 |
| COD | 455.51 | 3663.21 | NOR | -4480.09 | -39.51 |
| COL | 87908.66 | 239547.29 | NZL | 6805.27 | 310.17 |
| COM | 48.53 | 330.67 | OMN | 0.18 | 2.25 |
| CRI | 10849.88 | 4060.17 | PAK | 27478.59 | 323240.73 |
| CUB | 5362.37 | 59800.32 | PAN | 2963.11 | 59816.70 |
| CYP | 2670.77 | 52.91 | PER | 205.56 | 6889.60 |
| DEU | 74793.51 | 7820.51 | PHL | 183014.28 | 2000455.97 |
| DJI | 0.06 | 0.43 | PLW | 288.57 | 17872.01 |

| DMA | 1528.62 | 16489.54 | PNG | 3839.83 | 64083.53 |
| --- | --- | --- | --- | --- | --- |
| DNK | 3579.28 | 664.29 | POL | 15095.78 | 7.31 |
| DOM | 610.39 | 6624.28 | PRK | 33.30 | 262.58 |
| DZA | 128.15 | 1097.01 | PRT | 15736.01 | 132.71 |
| ECU | 19280.44 | 26076.81 | QAT | 99757.97 | 45507.20 |
| EGY | 6085.73 | 136417.16 | ROU | 7686.69 | 109.49 |
| ERI | 4.49 | 25.80 | RUS | -102770.71 | -252356.02 |
| ESP | 109318.39 | 33397.02 | SAU | 329374.08 | 39570906.40 |
| EST | -570.87 | -0.52 | SDN | 39789.78 | 270604.47 |
| FIN | -11633.24 | -143.76 | SEN | 2375.09 | 41178.03 |
| FJI | 40.36 | 408.02 | SGP | 62691.62 | 2871.16 |
| FRA | 120346.20 | 54059.76 | SLB | 26.93 | 369.21 |
| FSM | 613.50 | 5561.15 | SLE | 994.97 | 9272.77 |
| GAB | 213.53 | 2823.92 | SLV | 90.60 | 875.68 |
| GBR | 70410.65 | 3434.03 | SOM | 0.45 | 2.96 |
| GHA | 2894.53 | 70402.28 | STP | 0.00 | 0.00 |
| GIN | 52237.70 | 386764.64 | SUR | 20.26 | 599.23 |
| GMB | 84.43 | 1279.70 | SVN | 1588.51 | 1.19 |
| GNB | 661.54 | 5184.75 | SWE | -3627.51 | -44.33 |
| GNQ | 81.21 | 661.01 | SYC | 62691.62 | 11.69 |
| GRC | 21007.87 | 3868.22 | TGO | 137.40 | 995.84 |
| GRD | 30.30 | 332.16 | THA | 122974.57 | 394691.27 |
| GTM | 170.31 | 2010.03 | TLS | 88.99 | 698.96 |
| GUY | 2.67 | 99.37 | TON | 15165.63 | 129889.32 |
| HND | 2613.17 | 22162.64 | TTO | 15.08 | 156.38 |
| HRV | 3733.30 | 306.51 | TUN | 3700.17 | 40056.04 |
| HTI | 347.67 | 2281.88 | TUR | 108353.79 | 429.27 |
| IDN | 249459.29 | 10448681.62 | TZA | 1215.33 | 16077.05 |
| IND | 1632355.62 | 5267957.23 | UKR | 1731.25 | 37216.06 |
| IRL | 3012.94 | 14.66 | URY | 0.14 | 6.62 |
| IRN | 13250.60 | 152472.33 | USA | 1799000.95 | 60144937.93 |
| ISL | -1294.88 | -1.52 | VCT | 0.01 | 0.20 |
| ISR | 73710.00 | 353.63 | VEN | 40288.38 | 364591.87 |
| ITA | 105400.74 | 74099.55 | VNM | 3448.41 | 71446.53 |
| JAM | 25.68 | 643.76 | VUT | 16.06 | 158.26 |
| JPN | 207665.43 | 13600.54 | WSM | 5.57 | 51.04 |
| KEN | 785.98 | 8148.74 | YEM | 81483.09 | 771959.29 |
| KHM | 501.21 | 5040.66 | ZAF | 83078.12 | 508.18 |

**Table. S6: Linear mixed models to investigate the relationship between the ecosystem area and the three subsystems**

| Num  ber | Model | df | AIC | Conditional  R2 | Marginal  R2 |
| --- | --- | --- | --- | --- | --- |
| 1 | Rate ~x2 + x1+ x3 + (1\|nation) | 6 | -6724.640 | 0.871 | 0.064 |
| 2 | Rate ~lnx1 + lnx2 + lnx3+(1\|nation) | 6 | -6775.090 | 0.867 | 0.045 |
| 3 | Rate ~lnx1 + lnx2 + (1\|nation) | 5 | -6779.504 | 0.860 | 0.020 |
| 4 | Rate ~lnx3 + lnx2 + (1\|nation) | 5 | -6785.070 | 0.866 | 0.044 |
| **5** | **Rate ~lnx2 + (1\|nation)** | **4** | **-6789.703** | **0.859** | **0.018** |

Where *Rate* -growth rate of the blue carbon area over the next four years, x1-driving force, x2- development capacity, x3-resource endowment.

**Table. S7: Fixed effects of Model 5:**

|  | Estimate | Std.Error | df | t value | p value |
| --- | --- | --- | --- | --- | --- |
| (Intercept) | -0.01170 | 0.004265 | 113.2 | -2.744 | 0.00707 (<0.01) |
| lnx2 | 0.005357 | 0.000695 | 1275 | 7.707 | 2.57e-14 (<0.001) |

A t-test was used, and it was two-sided; no adjustments were made for multiple comparisons.

**Table. S8: Random effects Model 5:**

| Groups Name | Variance | Std.Dev. |
| --- | --- | --- |
| nation (Intercept) | 0.001483 | 0.03851 |
| Residual | 0.0002494 | 0.01579 |

Number of obs: 1325, groups: nation, 94

**Table. S9. ISO3 code details and regions. The division of regions is based on the United Nations Sustainable Development Goals.**

| **ISO3** | **Nation** | **Region** | **ISO3** | **Nation** | **Region** |
| --- | --- | --- | --- | --- | --- |
| AGO | Angola | Sub-Saharan Africa | KIR | Kiribati | Oceania |
| ALB | Albania | Europe and Northern America | KNA | Saint Kitts and Nevis | Latin America and the Caribbean |
| ARE | United Arab Emirates | Northern Africa and Western Asia | KWT | Kuwait | Northern Africa and Western Asia |
| ARG | Argentina | Latin America and the Caribbean | LBR | Liberia | Sub-Saharan Africa |
| ATG | Antigua and Barbuda | Latin America and the Caribbean | LBY | Libya | Northern Africa and Western Asia |
| AUS | Australia | Oceania | LCA | Saint Lucia | Latin America and the Caribbean |
| BEL | Belgium | Europe and Northern America | LKA | Sri Lanka | Central and Southern Asia |
| BEN | Benin | Sub-Saharan Africa | LVA | Latvia | Europe and Northern America |
| BGD | Bangladesh | Central and Southern Asia | MDG | Madagascar | Sub-Saharan Africa |
| BGR | Bulgaria | Europe and Northern America | MDV | Maldives | Central and Southern Asia |
| BHR | Bahrain | Northern Africa and Western Asia | MEX | Mexico | Latin America and the Caribbean |
| BHS | Bahamas | Latin America and the Caribbean | MHL | Marshall Islands | Oceania |
| BIH | Bosnia and Herzegovina | Europe and Northern America | MLT | Malta | Europe and Northern America |
| BLZ | Belize | Latin America and the Caribbean | MMR | Myanmar | Eastern and South-Eastern Asia |
| BRA | Brazil | Latin America and the Caribbean | MNE | Montenegro | Europe and Northern America |

| BRB | Barbados | Latin America and the Caribbean | MOZ | Mozambique | Sub-Saharan Africa |
| --- | --- | --- | --- | --- | --- |
| BRN | Brunei | Eastern and South-Eastern Asia | MRT | Mauritania | Sub-Saharan Africa |
| CAN | Canada | Europe and Northern America | MUS | Mauritius | Sub-Saharan Africa |
| CHL | Chile | Latin America and the Caribbean | MYS | Malaysia | Eastern and South-Eastern Asia |
| CHN | China | Eastern and South-Eastern Asia | NGA | Nigeria | Sub-Saharan Africa |
| CIV | Ivory Coast | Sub-Saharan Africa | NIC | Nicaragua | Latin America and the Caribbean |
| CMR | Cameroon | Sub-Saharan Africa | NLD | Netherlands | Europe and Northern America |
| COD | Democratic Republic of the  Congo | Sub-Saharan Africa | NOR | Norway | Europe and Northern America |
| COL | Colombia | Latin America and the Caribbean | NZL | New Zealand | Oceania |
| COM | Comoro Islands | Sub-Saharan Africa | OMN | Oman | Northern Africa and Western Asia |
| CRI | Costa Rica | Latin America and the Caribbean | PAK | Pakistan | Central and Southern Asia |
| CUB | Cuba | Latin America and the Caribbean | PAN | Panama | Latin America and the Caribbean |
| CYP | Cyprus | Northern Africa and Western Asia | PER | Peru | Latin America and the Caribbean |
| DEU | Germany | Europe and Northern America | PHL | Philippines | Eastern and South-Eastern Asia |
| DJI | Djibouti | Sub-Saharan Africa | PLW | Palau | Oceania |
| DMA | Dominica | Latin America and the Caribbean | PNG | Papua New Guinea | Oceania |
| DNK | Denmark | Europe and Northern America | POL | Poland | Europe and Northern America |
| DOM | Dominican Republic | Latin America and the Caribbean | PRK | North Korea | Eastern and South-Eastern Asia |
| DZA | Algeria | Northern Africa and Western Asia | PRT | Portugal | Europe and Northern America |
| ECU | Ecuador | Latin America and the Caribbean | QAT | Qatar | Northern Africa and Western Asia |
| EGY | Egypt | Northern Africa and Western Asia | ROU | Romania | Europe and Northern America |
| ERI | Eritrea | Sub-Saharan Africa | RUS | Russia | Europe and Northern America |
| ESP | Spain | Europe and Northern America | SAU | Saudi Arabia | Northern Africa and Western Asia |
| EST | Estonia | Europe and Northern America | SDN | Sudan | Northern Africa and Western Asia |
| FIN | Finland | Europe and Northern America | SEN | Senegal | Sub-Saharan Africa |
| FJI | Fiji | Oceania | SGP | Singapore | Eastern and South-Eastern Asia |
| FRA | France | Europe and Northern America | SLB | Solomon Islands | Oceania |
| FSM | Micronesia | Oceania | SLE | Sierra Leone | Sub-Saharan Africa |
| GAB | Gabon | Sub-Saharan Africa | SLV | El Salvador | Latin America and the Caribbean |
| GBR | United Kingdom | Europe and Northern America | SOM | Somalia | Sub-Saharan Africa |
| GHA | Ghana | Sub-Saharan Africa | STP | Sao Tome and  Principe | Sub-Saharan Africa |
| GIN | Guinea | Sub-Saharan Africa | SUR | Suriname | Latin America and the Caribbean |
| GMB | Gambia | Sub-Saharan Africa | SVN | Slovenia | Europe and Northern America |
| GNB | Guinea Bissau | Sub-Saharan Africa | SWE | Sweden | Europe and Northern America |
| GNQ | Equatorial Guinea | Sub-Saharan Africa | SYC | Seychelles | Sub-Saharan Africa |
| GRC | Greece | Europe and Northern America | TGO | Togo | Sub-Saharan Africa |
| GRD | Grenada | Latin America and the Caribbean | THA | Thailand | Eastern and South-Eastern Asia |
| GTM | Guatemala | Latin America and the Caribbean | TLS | East Timor | Eastern and South-Eastern Asia |
| GUY | Guyana | Latin America and the Caribbean | TON | Tonga | Oceania |
| HND | Honduras | Latin America and the Caribbean | TTO | Trinidad and Tobago | Latin America and the Caribbean |
| HRV | Croatia | Europe and Northern America | TUN | Tunisia | Northern Africa and Western Asia |

| HTI | Haiti | Latin America and the Caribbean | TUR | Turkey | Northern Africa and Western Asia |
| --- | --- | --- | --- | --- | --- |
| IDN | Indonesia | Eastern and South-Eastern Asia | TZA | Tanzania | Sub-Saharan Africa |
| IND | India | Central and Southern Asia | UKR | Ukraine | Europe and Northern America |
| IRL | Ireland | Europe and Northern America | URY | Uruguay | Latin America and the Caribbean |
| IRN | Iran | Central and Southern Asia | USA | United States | Europe and Northern America |
| ISL | Iceland | Europe and Northern America | VCT | Saint Vincent and the  Grenadines | Latin America and the Caribbean |
| ISR | Israel | Northern Africa and Western Asia | VEN | Venezuela | Latin America and the Caribbean |
| ITA | Italy | Europe and Northern America | VNM | Vietnam | Eastern and South-Eastern Asia |
| JAM | Jamaica | Latin America and the Caribbean | VUT | Vanuatu | Oceania |
| JPN | Japan | Eastern and South-Eastern Asia | WSM | Samoa | Oceania |
| KEN | Kenya | Sub-Saharan Africa | YEM | Yemen | Northern Africa and Western Asia |
| KHM | Cambodia | Eastern and South-Eastern Asia | ZAF | South Africa | Sub-Saharan Africa |

**Reference**

1. Mohsin, M. et al. Developing low carbon economies: An aggregated composite index based on carbon emissions. Sustainable Energy Technologies and Assessments 35, 365–374 (2019).
2. Xu, Z. et al. Assessing progress towards sustainable development over space and time. Nature 577, 74–78 (2020).
3. Friedlingstein, P. et al. Global Carbon Budget 2022. Earth Syst. Sci. Data 14, 4811–4900 (2022).
4. World Bank. Indicators | Data. https://data.worldbank.org/indicator/.
5. Tan, S. et al. A holistic low carbon city indicator framework for sustainable development. Applied Energy 185, 1919–1930 (2017).
6. Looney, B. Statistical Review of World Energy, bp global https://[www.bp.com/en/global/corporate/energy-](http://www.bp.com/en/global/corporate/energy-) economics/statistical-review-of-world-energy.html. (2020).
7. Berrang-Ford, L. et al. What drives national adaptation? A global assessment. Climatic Change 124, 441–450 (2014).
8. Eckstein, D., Künzel, V. & Schäfer, L. Global Climate Risk Index 2021. Germanwatch e.V. https://[www.germanwatch.org/en/19777](http://www.germanwatch.org/en/19777) (2021).
9. Peters, G. P. et al. Key indicators to track current progress and future ambition of the Paris Agreement. Nature Clim Change 7, 118–122 (2017).
10. United Nations Treaty Collection. https://treaties.un.org/Pages/ViewDetails.aspx?src=TREATY&mtdsg_no=XXVII-7-a&chapter=27&clang=_en.
11. United Nations Treaty Collection. https://treaties.un.org/Pages/ViewDetails.aspx?src=TREATY&mtdsg_no=XXVII-7- d&chapter=27&clang=_en.
12. Perdan, S. & Azapagic, A. Carbon trading: Current schemes and future developments. Energy Policy 39, 6040– 6054 (2011).
13. Jung, H. & Song, C.-K. Effects of emission trading scheme (ETS) on change rate of carbon emission. Sci Rep 13, 912 (2023).
14. International Carbon Action Partnership (ICAP). https://icapcarbonaction.com/node/836 (2023).
15. Andrijevic, M., Crespo Cuaresma, J., Muttarak, R. & Schleussner, C.-F. Governance in socioeconomic pathways and its role for future adaptive capacity. Nat Sustain 3, 35–41 (2020).
16. Transparency International. Corruption Perceptions Index: Transparency.org. https://[www.transparency.org/en/cpi/2022.](http://www.transparency.org/en/cpi/2022)
17. Kelly, C., Ellis, G. & Flannery, W. Conceptualising change in marine governance: Learning from Transition Management. Marine Policy 95, 24–35 (2018).
18. OECD, Organization for Economic Co-operation and Development. Sustainable Ocean Economy. https://stats.oecd.org/index.aspx?datasetcode=OCEAN.
19. United Nations. The Global Ocean Science Report 2020. https://gosr.ioc-unesco.org/home.
20. SCImago. Scimago Journal & Country Rank. https://[www.scimagojr.com/.](http://www.scimagojr.com/)
21. Mascia, M. B. et al. A novel framework for analyzing conservation impacts: evaluation, theory, and marine protected areas. Annals of the New York Academy of Sciences 1399, 93–115 (2017).
22. UNEP & IUCN. Protected Planet: [marine-protected-areas; The World Database on Protected Areas (WDPA)/Database on other effective area-based conservation measures] [On-line], [April/2021], Cambridge, UK: UNEP-WCMC and IUCN. https://[www.protectedplanet.net/en/thematic-areas/marine-protected-areas.](http://www.protectedplanet.net/en/thematic-areas/marine-protected-areas) (2021)
23. Fox, H. E. et al. How Are Our MPAs Doing? Challenges in Assessing Global Patterns in Marine Protected Area Performance. Coastal Management 42, 207–226 (2014).
24. Gill, D. A. et al. Capacity shortfalls hinder the performance of marine protected areas globally. Nature 543, 665– 669 (2017).
25. Richards, D. R., Thompson, B. S. & Wijedasa, L. Quantifying net loss of global mangrove carbon stocks from 20 years of land cover change. Nat Commun 11, 4260 (2020).
26. Bunting, P. et al. The Global Mangrove Watch—A New 2010 Global Baseline of Mangrove Extent. Remote Sensing 10, 1669 (2018).
27. Central Intelligence Agency. The CIA World Factbook 2021-2022. (2021).
28. Cisneros-Montemayor, A. M. et al. Enabling conditions for an equitable and sustainable blue economy. Nature 591, 396–401 (2021).
29. Mcowen, C. et al. A global map of saltmarshes. Biodiversity Data Journal 5, e11764 (2017).
30. UNEP-WCMC & Short, F. T. Global Distribution of Seagrasses. Seventh update to the data layer used in Green and Short (2003). Cambridge (UK): UN Environment World Conservation Monitoring Centre. [http://data.unep-](http://data.unep-/) wcmc.org/datasets/7.
31. Halpern, B. S. et al. An index to assess the health and benefits of the global ocean. Nature 488, 615–620 (2012).
32. Ouyang, X. & Lee, S. Y. Improved estimates on global carbon stock and carbon pools in tidal wetlands. Nat Commun 11, 317 (2020).
33. Wang, F. et al. Global blue carbon accumulation in tidal wetlands increases with climate change. National Science Review 8, nwaa296 (2021).
34. Mcleod, E. et al. A blueprint for blue carbon: toward an improved understanding of the role of vegetated coastal habitats in sequestering CO2. Frontiers in Ecology and the Environment 9, 552–560 (2011).
