# Supplementary material for: Sustainably developing global blue carbon for climate change mitigation and economic benefits through international cooperation
Source: Nat Commun. 2023 Oct 2;14:6144. doi: 10.1038/s41467-023-41870-x (PMC10545692; doi:10.1038/s41467-023-41870-x)
Supplement: Supplementary file 2 — Reporting Summary [file 41467_2023_41870_MOESM2_ESM.pdf]

nature portfolio

Corresponding author(s): Guanqiong Ye and Zhenci Xu

Last updated by author(s): Jul 22, 2023

Reporting Summary

Nature Portfolio wishes to improve the reproducibility of the work that we publish. This form provides structure for consistency and transparency in reporting. For further information on Nature Portfolio policies, see our Editorial Policies and the Editorial Policy Checklist.

Statistics

For all statistical analyses, confirm that the following items are present in the figure legend, table legend, main text, or Methods section.

n/a	Confirmed	
☒	☐	The exact sample size (*n*) for each experimental group/condition, given as a discrete number and unit of measurement
☒	☐	A statement on whether measurements were taken from distinct samples or whether the same sample was measured repeatedly
☐	☒	The statistical test(s) used AND whether they are one- or two-sided<br>*Only common tests should be described solely by name; describe more complex techniques in the Methods section.*
☒	☐	A description of all covariates tested
☒	☐	A description of any assumptions or corrections, such as tests of normality and adjustment for multiple comparisons
☐	☒	A full description of the statistical parameters including central tendency (e.g. means) or other basic estimates (e.g. regression coefficient) AND variation (e.g. standard deviation) or associated estimates of uncertainty (e.g. confidence intervals)
☐	☒	For null hypothesis testing, the test statistic (e.g. *F*, *t*, *r*) with confidence intervals, effect sizes, degrees of freedom and *P* value noted<br>*Give P values as exact values whenever suitable.*
☒	☐	For Bayesian analysis, information on the choice of priors and Markov chain Monte Carlo settings
☒	☐	For hierarchical and complex designs, identification of the appropriate level for tests and full reporting of outcomes
☐	☒	Estimates of effect sizes (e.g. Cohen's *d*, Pearson's *r*), indicating how they were calculated

Our web collection on statistics for biologists contains articles on many of the points above.

Software and code

Policy information about availability of computer code

Data collection	We obtained the data in this study from the following sources: United Nations, UN Environment Programme, World Bank, Transparency International, Organisation for Economic Co-operation and Development (OECD), International Carbon Action Partnership, and literature.
Data analysis	We use Microsoft Excel 2016, ArcGIS10.7, and R-4.0.5 to analyze data.

For manuscripts utilizing custom algorithms or software that are central to the research but not yet described in published literature, software must be made available to editors and reviewers. We strongly encourage code deposition in a community repository (e.g. GitHub). See the Nature Portfolio guidelines for submitting code & software for further information.

Data

Policy information about availability of data

All manuscripts must include a data availability statement. This statement should provide the following information, where applicable:

- Accession codes, unique identifiers, or web links for publicly available datasets
- A description of any restrictions on data availability
- For clinical datasets or third party data, please ensure that the statement adheres to our policy

All the sources of data underlying the research are available in the Supplementary Information. All other data are available from the corresponding authors upon reasonable request.

Research involving human participants, their data, or biological material

Policy information about studies with human participants or human data. See also policy information about sex, gender (identity/presentation), and sexual orientation and race, ethnicity and racism.

Reporting on sex and gender	We didn't collect information about human research participants and Sex and Gender.
Reporting on race, ethnicity, or other socially relevant groupings	We didn't collect information about race, ethnicity, or other socially relevant groupings.
Population characteristics	We didn't collect information about human research participants.
Recruitment	We didn't collect information about recruitment.
Ethics oversight	We didn't collect information about human research and no ethical oversight

Note that full information on the approval of the study protocol must also be provided in the manuscript.

Field-specific reporting

Please select the one below that is the best fit for your research. If you are not sure, read the appropriate sections before making your selection.

☐ Life sciences ☐ Behavioural & social sciences ☒ Ecological, evolutionary & environmental sciences

For a reference copy of the document with all sections, see nature.com/documents/nr-reporting-summary-flat.pdf

Ecological, evolutionary & environmental sciences study design

All studies must disclose on these points even when the disclosure is negative.

Study description	Blue carbon is adopted as a global climate change solution. Mangrove, salt marsh, and seagrass as the three blue carbon ecosystems are also gaining traction internationally because of their role in coastal social welfare growth. However, the global assessment of sustainable development level of blue carbon has not been conducted. Here, we constructed a blue carbon development index (BCDI) system, comprising three subsystems: driving force, resource endowment, and development capacity, to assess the sustainable development level of 136 coastal countries' blue carbon over 24 consecutive years. Results showed an upward trend in BCDI scores across nations, with regional performance imbalance, and we found a positive correlation between development capacity and blue carbon resource endowment. Based on the scenario simulations of global cooperation, we found that coastal countries could improve the global average BCDI score, add 2,96 Mt of annual carbon sequestration, and generate $136.34 million in 2030 under Global Deep Cooperation scenario compared with the Business-As-Usual scenario.<br>Our study advanced the knowledge in understanding long-term sustainable development status of global blue carbon across coastal countries, and could provide useful information for international blue carbon cooperations. Our results encourage coastal countries to conserve blue carbon ecosystems through international cooperation for co-improving carbon sequestration capacity and multiple social welfares.
Research sample	We sampled the coastal countries with blue carbon ecosystems and collected data at a national scale by considering the blue carbon resources, economy, technology, ecology, and responsibility for reducing emissions. We established an index system called BCDI by setting with three sub-systems, namely driving force, development capacity, resource endowment, and 18 specific indicators. The data were collected from World bank, United Nations, Protected Planet of UNEP, OECD, Global Carbon Atlas, Statistical Review of World Energy, Transparency International, International Carbon Action Partnership, and literature. See details in the Methods section.
Sampling strategy	We focused on the three blue carbon ecosystems with no scientific controversy based on several guidelines from UNEP, IUCN, IPCC, etc. We then searched the coastal countries/nations and pinpointed 136 coastal countries with recorded blue carbon ecosystems from the UNEP's distribution data sets of the three blue carbon ecosystems, namely mangroves, salt marsh, and seagrass beds.
Data collection	We acquired the data in this study from the following sources: United Nations, UN Environment Programme, World Bank, Transparency International, Organisation for Economic Co-operation and Development (OECD), International Carbon Action Partnership, and literature. See Data Sources in the Methods section for details.
Timing and spatial scale	We studied 136 coastal countries with blue carbon ecosystems at a national scale from 1996 to 2019
Data exclusions	No data was excluded
Reproducibility	We have provided detailed descriptions of the calculation methods, data sources, and models. Besides, we commit to making the data, models, code, etc., used in our research available to those interested so that others can use the data to reproduce our results.
Randomization	We study coastal countries with blue carbon ecosystems. These countries are extracted from the UNEP's distribution data sets of mangroves, salt marsh, and seagrass beds. See Data Sources in the Methods section.

Blinding	No blinding. We study coastal countries with blue carbon ecosystems

Did the study involve field work? ☐ Yes ☒ No

Reporting for specific materials, systems and methods

We require information from authors about some types of materials, experimental systems and methods used in many studies. Here, indicate whether each material, system or method listed is relevant to your study. If you are not sure if a list item applies to your research, read the appropriate section before selecting a response.

Materials & experimental systems

n/a	Involved in the study
☒	☐ Antibodies
☒	☐ Eukaryotic cell lines
☒	☐ Palaeontology and archaeology
☒	☐ Animals and other organisms
☒	☐ Clinical data
☒	☐ Dual use research of concern
☒	☐ Plants

Methods

n/a	Involved in the study
☒	☐ ChIP-seq
☒	☐ Flow cytometry
☒	☐ MRI-based neuroimaging
